# AAV-mediated genome targeting of the *GLA* cDNA in newborn and juvenile *Gla* KO mice achieves long-term correction of Fabry disease

**DOI:** 10.1016/j.omta.2026.201864

**Published:** 2026-10-15

**Authors:** Himanshi Saxena, Giulia Romano, Rossana Domenis, Jessica Biasizzo, Martina Ferro, Dania Ferino, Antonio Vicidomini, Andrea Colliva, Alessandra Iaconcig, Giulia Bortolussi, Lorena Zentilin, Andrea Dardis, Andrés F. Muro

**Affiliations:** 1 International Centre for Genetic Engineering and Biotechnology, Padriciano, 99, 34149 Trieste, Italy; 2 Institute of Clinical Pathology, Department of Laboratory Medicine, University Hospital of Udine, P. Le Santa Maria Della Misericordia 15, 33100 Udine, Italy; 3 Regional Coordinator Centre for Rare Diseases, University Hospital of Udine, P. Le Santa Maria Della Misericordia 15, 33100 Udine, Italy

**Keywords:** early-onset Fabry disease, gene therapy, mouse model, enzyme replacement therapy, long-term treatment, hot-plate test

## Abstract

Fabry disease (FD) is an X-linked lysosomal storage disorder caused by mutations in the *GLA* gene, resulting in deficient α-galactosidase A (α-Gal A) activity and progressive substrate accumulation in multiple organs. Current enzyme replacement therapies are limited by short enzyme half-life, immunogenicity, and high costs, underscoring the need for durable treatments, particularly in the context of newborn screening programs that enable early diagnosis. Here, we employed AAV-mediated genome targeting to insert a promoter-less codon-optimized human *GLA* cDNA into the albumin locus of Fabry mice, with homologous recombination enhanced by SaCas9 nuclease or fludarabine. This approach permanently modifies the hepatocyte genomes, converting them into bio-factories that secrete α-Gal A, which is then captured by organs expressing mannose-6-phosphate receptors, reducing glycosphingolipid accumulation in affected tissues. In fact, treatment of newborn and juvenile mice resulted in sustained supraphysiological α-Gal A activity, complete or near-complete clearance of lyso-Gb3 in plasma and target tissues, and prevention of neuropathic deficits, as assessed by the hot-plate test. These findings demonstrate that hepatocyte genome editing can provide a one-time, long-lasting therapeutic strategy for FD, potentially applicable across diverse *GLA* mutations and suitable for early intervention in patients identified through newborn screening, aiming at improving patient outcomes.

## Graphical abstract

**Figure:**
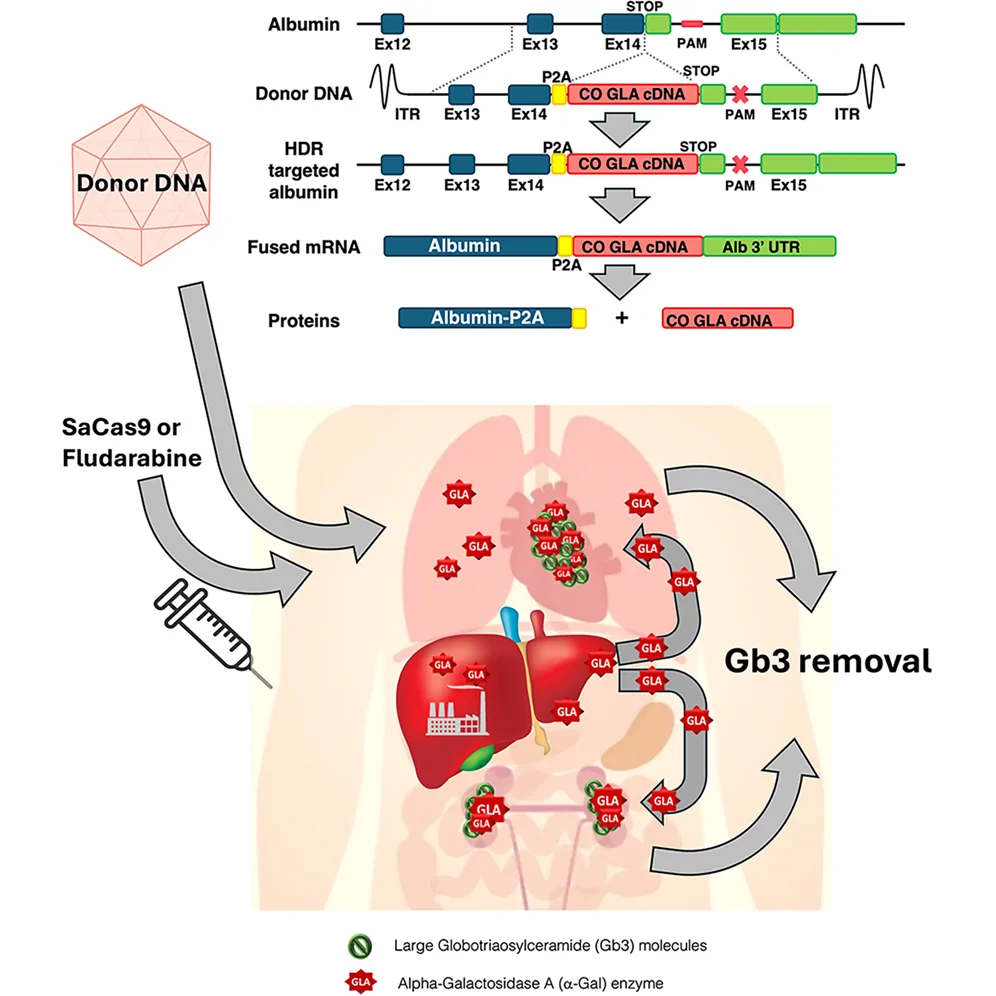

## Teaser

Saxena and colleagues tested a gene targeting strategy for Fabry disease based on the permanent insertion of a codon-optimized GLA cDNA targeting the albumin gene in mice. The enzyme produced by hepatocytes is secreted into circulation and captured by target organs, preventing accumulation of toxic metabolites and organ damage.

## Introduction

Fabry disease (FD) is an X-linked lysosomal storage disorder caused by mutations in the *GLA* gene and consequent deficient activity of the lysosomal α-galactosidase A (α-Gal A) enzyme, leading to accumulation of glycosphingolipids in lysosomes, such as globotriaosylceramide (Gb3) and its deacylated form (globotriaosylsphingosine, lyso-Gb3), causing progressive multi-organ failure.^1^^,^^2^^,^^3^ Prevalence ranges from 1/3,100 to 1/117,000 males in Europe,^1^ with higher frequencies found in newborn screenings: 1 in 1,368 affected males in Taiwan^4^ and 1 in 3,100 to 1 in 4,121 affected males in Italy.^5^^,^^6^ The classical FD phenotype, featuring minimal α-Gal A enzyme activity, manifests during childhood with extremity and abdominal pain, diarrhea, sweating abnormalities, recurrent fever, fatigue, cornea verticillata, and angiocheratomas. The late-onset FD presents with higher residual enzyme activity and usually has later manifestations that might affect a single organ.^1^^,^^2^^,^^3^ Life expectancy is reduced by 18 years in males and 5 years in females.^7^

Currently, the standard of care is enzyme replacement therapy (ERT), which restores deficient α-Gal A to reduce glycosphingolipid accumulation in tissues. However, this therapeutic strategy presents several limitations and high costs (∼€200k/year/patient).^8^^,^^9^^,^^10^ In some patients, correction is very limited with poor clearance of substrates in target organs. The half-life of the enzyme is very short,^11^^,^^12^^,^^13^ and administration requires 2- to 4-h bi-weekly intravenous infusions. About 40% of patients develop neutralizing anti-drug antibodies (ADA), reducing therapeutic efficacy.^14^ A second-generation ERT has been recently approved (pegunigalsidase alfa, PEGylated, Chiesi, Parma, Italy), which is expected to increase the circulating half-life and reduce immunogenicity.^15^^,^^16^^,^^17^ Other treatments, such as chaperone administration, aim to stabilize mutant α-Gal A and reduce Gb3 production,^18^ but they can only be applied to patients with “amenable” mutations in the *GLA* gene (some mutations resulting in late onset FD), while substrate reduction therapy (SRT) has limitations and may require the combination with ERT.^19^ Hence, there is a clinical need for more effective and long-lasting therapies that not only alleviate disease-related symptoms but also improve the quality of life for patients with FD. Newborn screening is already implemented in some regions of Italy, in Taiwan, and in some states of the United States, while other countries are conducting pilot studies.^20^^,^^21^ Due to the expected very early diagnosis, these new therapies should also be applicable to very young patients of FD.

Gene therapy and genome editing approaches are promising approaches expected to result in long-term therapeutic correction.^22^^,^^23^ Gene therapy approaches with recombinant adeno-associated vectors (rAAV) targeting the liver have demonstrated encouraging results in both clinical and preclinical investigations of various inherited diseases, including adult patients of FD.^24^^,^^25^^,^^26^^,^^27^^,^^28^^,^^29^^,^^30^^,^^31^^,^^32^^,^^33^ The liver is a strategic organ for gene therapy approaches due to its tolerogenic properties,^34^^,^^35^ which reduce the risk of the formation of ADA. In fact, AAV-mediated liver gene therapy has shown effective α-Gal A production and antigen-specific tolerance in adult FD mice, reducing the risk of ADA formation.^26^^,^^27^ Ongoing clinical trials aim at enhancing α-Gal A production and assessing long-term safety and efficacy.

With the implementation of neonatal screening for FD, patients may be diagnosed and treated before the onset of the first symptoms.^20^^,^^36^^,^^37^^,^^38^ However, in children, hepatocyte proliferation during liver growth may cause the gradual loss of the episomal AAV DNA, leading to diminished therapeutic efficacy and the need for re-dosing.^39^^,^^40^^,^^41^ Re-administration is limited by the presence of anti-AAV neutralizing antibodies (Nab) generated during the first AAV administration,^33^^,^^42^^,^^43^ and strategies to deplete Nab are under investigation, though they require clinical validation.^44^

Genome editing offers the potential for a single dosing with a long-term cure by directly correcting the *GLA* mutation.^22^^,^^23^ For instance, a study successfully corrected a 146G>A mutation *in vitro* using adenine base editors, restoring α-Gal A activity.^45^ While promising, this approach requires mutation-specific strategies, posing significant limitations to its translation into the clinic. mRNA therapies are also being explored as a short-term solution, delivering *GLA* mRNA to cells for protein production.^46^ However, mRNA therapy would require frequent injections, with a significant increase in the costs and limiting its long-term practicality.

We here propose a genome targeting strategy based on the insertion of a promoterless codon-optimized human *GLA* cDNA into the albumin locus.^47^ To enhance the gene targeting rate, we used SaCas9 and a sgRNA targeting the intron downstream of the stop codon-containing exon of the albumin gene^48^ or fludarabine, an FDA-approved compound that induces transient DNA damage signaling, enhancing AAV-homologous recombination in hepatocytes.^49^ We have previously tested these strategies in different models of disease, showing its efficacy for cytoplasmic, ER-bound, and secreted proteins resulting in the full rescue of lethality and normalization of the phenotypes.^48^^,^^49^^,^^50^^,^^51^^,^^52^

Our strategy aims at providing a one-time, permanent cure by genetically modifying the hepatocyte genome to produce high levels of α-Gal A. We show that the secreted, circulating α-Gal A is captured by affected tissues, such as ERT, with reversal of the FD phenotype. Treatment of newborn *Gla* KO mice with AAV-mediated genome targeting into the albumin locus prevents lyso-Gb3 accumulation in target organs and the development of the neurological phenotype.

## Results

### Genome targeting at different neonatal ages

To determine the efficacy of the genome targeting strategy at different post-natal ages, we first performed a time-response curve by treating C57Bl/6 WT male mice at different post-natal ages (P1, P5, P10, P15, P20, and P30; Figure 1A) with 3.0E14 vg/kg for AAV8-pAB288-Alb-EGFP (Donor vector) and pX602-sgRNA-Cas9 (1:1; Donor: Cas9).^48^^,^^51^^,^^52^ The donor vector is designed to insert the eGFP transgene, preceded by an in-frame 2A peptide (P2A), by the homology-directed repair mechanism, just before the natural albumin stop codon.^47^^,^^48^^,^^50^ The robust albumin promoter transcribes a chimeric mRNA that is translated into two proteins: albumin and eGFP. As a comparison group, mice were treated with 3.0E12 vg/kg with AAV8-pGG2-AAT-eGFP, a non-integrative vector. This dose provides full and long-term correction to Fabry mice when a similar vector with the h*GLA* cDNA is delivered at P30, by preventing lyso-Gb3 accumulation in plasma and target tissues, and fully correcting neuropathic involvement.^53^ All animals were sacrificed at 2 months of age, and the liver was harvested for histological and eGFP-expression analysis. None of the treatments affected the growth curve of the animals (Figure S1). In the genome targeting groups, the eGFP signal in the western blot of liver protein extracts was higher at early timepoints and declined in the following timepoints, with a slight increase at P30 (Figures 1B and 1C). On the contrary, in the episomal groups, the signal was barely detected at the early timepoints and increased in the later timepoints, peaking in the group treated at P30. These results were confirmed by the quantification of eGFP-positive hepatocytes in liver sections (Figures 1D and 1E).

**Figure 1:**
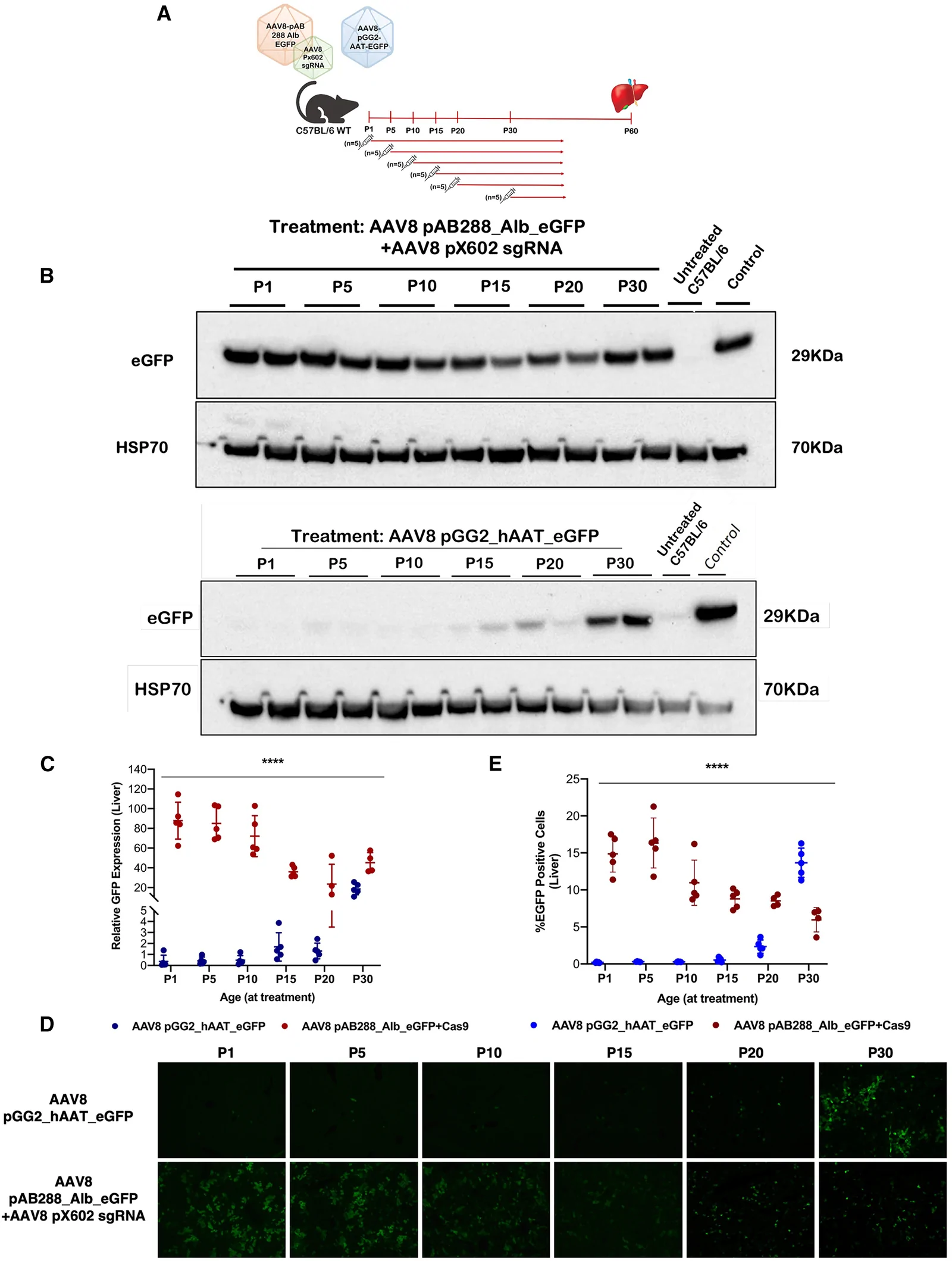
Comparison in the liver of episomal vs. integrating AAV-mediated approaches at different post-natal ages (A) Experimental design. Mice were dosed at different ages with the episomal (AAV8 pGG2_hAAT-eGFP) or integrating AAV (AAV8 pAB288_Alb_eGFP and AAV8 pX602 sgRNA) vectors containing the eGFP cDNA, sacrificed at P60, and the liver collected for analysis. (B) Western blot analysis of liver protein extracts from the animals treated with the integrating (top) or the episomal (bottom) strategies. The housekeeping gene HSP70 was used as internal control. (C) Quantification of the eGFP WB shown in (B). Each dot corresponds to a single animal. Mixed-effects model (REML), ∗∗∗∗; time: *p* = 0.0001; treatment: *p* < 0.0001; time × treatment: *p* < 0.0001. (D) Immunofluorescence of representative liver sections of the different treated animals. (E) Quantification of the P60 liver sections. Each dot corresponds to the mean of 15–20 sections from a single animal. Mixed-effects model (REML), ∗∗∗∗; time: *p* = 0.0003; treatment: *p* < 0.0001; time × treatment: *p* < 0.0001.

### Genome targeting in juvenile Fabry mice results in almost complete lyso-Gb3 clearance

Based on the results of Figure 1 showing satisfactory genome targeting rate and transgene expression in the group treated at P30, we initially selected this time point, aiming at mimicking the treatment of juvenile patients of FD. Thus, we treated Fabry male mice with the donor vector containing a promoterless WT or codon-optimized h*GLA* cDNA flanked by albumin homology arms and the P2A sequence (Figure 2A; AAV8-Donor DNA 3.0E13 vg/kg). After successful genome targeting, the mature, glycosylated α-Gal A enzyme is secreted at high levels into the plasma for uptake by tissues via M6P-receptor-mediated uptake.^54^^,^^55^ To increase genome targeting rate, mice were co-injected with an AAV vector expressing the SaCas9 and an sgRNA targeting the albumin locus,^48^ or treated with fludarabine (Figures 2A and S2A).^49^ As a control, a group of mice were treated with the same dose of only the donor vector. Given the relatively high spontaneous recombination rate observed in P30 mice treated with the “donor-only” construct, we expected a significant substrate clearance in this group, with SaCas9 and fludarabine further increasing genome targeting efficiency^49^ and, consequently, lyso-Gb3 clearance. Blood samples were taken every 30 days for α-Gal A enzyme activity and lyso-Gb3 determination, mice were sacrificed at P150, and tissues were dissected for further analysis. The growth curve of all treated groups was like that of the WT and 129-Gla^tm1Kul/J^ mice, except the group treated with Donor CO02+Cas9 which showed increased weight (Figure S2B). While in the untreated mutant mice and in the WT groups, we observed a minor increase over time in the α-Gal A enzyme activity with constant lyso-Gb3 levels, in all treated groups, both α-Gal A enzyme activity and lyso-Gb3 levels in plasma were constant for the duration of the experiment (Figures 2B and 2C). In all cases, the enzyme activity of treated groups was higher than that of WT mice, although it reached statistical significance only for the groups treated with Donor DNA plus SaCas9 (Figures 2B–2D; Tables S1 and S2). The group of mice treated with Donor DNA and SaCas9 presented the highest plasma α-Gal A enzyme activity, followed by the groups Donor DNA and Fludarabine (Figures 2B–2D; Tables S1 and S2). The groups treated with only Donor DNA presented the lowest enzyme levels, although it was much higher than untreated mutant mice. The liver-secreted enzyme was captured by kidney and heart, being the groups treated with Donor DNA and Cas9 vectors the ones having the highest α-Gal A enzyme activity in these organs (Figures 2F–2H; Tables S1 and S2). Lyso-Gb3 levels in all treated groups were significantly lower than untreated 129-Gla^tm1Kul/J^ mice. Despite observing the highest enzyme activity in the groups treated with Donor DNA and Cas9 (Tukey’s comparison test, *p* < 0.001), the “Donor CO02+Cas9” group presented higher lyso-Gb3 levels than the mice treated with Donor DNA plus fludarabine (Tukey’s comparison test, *p* < 0.01, Table S2) and all other groups, except the “Donor WT + Cas9” group; while the difference in the “Donor WT + Cas9” versus “Donor plus fludarabine” did not reach statistical significance (Figures 2E and 2I–2K; Table S2). Unexpectedly, in the “Donor CO02+Cas9” group, we could not observe a clear correlation between enzyme activity, lyso-Gb3 levels, and gene targeting rate, although these differences were not statistically significant (Figures 2 and S2; Tables S1–S3).

**Figure 2:**
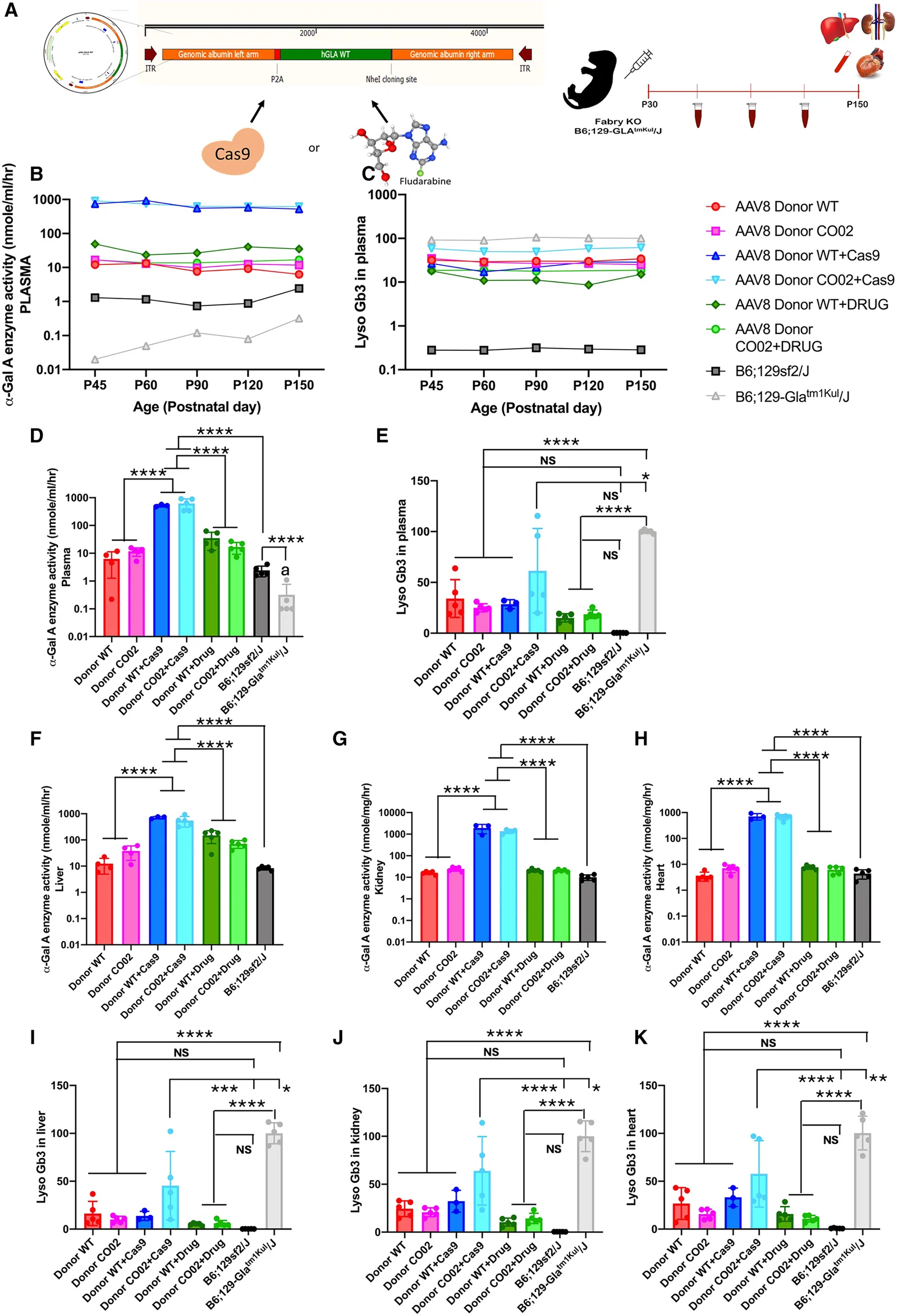
Genome targeting treatment of juvenile P30 Fabry mice (A) Experimental scheme. Postnatal day 30 (P30) male Fabry mice were treated with the donor vector (WT or CO02 cDNA versions; 3.0E13 vg/kg) alone, with fludarabine treatment, or co-infected with a second AAV vector carrying the SaCas9 cDNA and the sgRNA. (B) The α-Gal A enzyme activity in plasma is shown in the graph. (C) The relative lyso-Gb3 levels in plasma are shown in the graph. The levels present in Fabry mice correspond to 100%. (D and F–H) The α-Gal A enzyme activity at P150 in plasma (D), liver (F), kidney (G), and heart (H) are shown. One-way ANOVA (D, F, G, and H), *p* < 0.0001. (E and I–K) The lyso-Gb3 levels at P150 in plasma (E), liver (I), kidney (J), and heart (K) are shown. One-way ANOVA (E and I–K), ∗∗∗∗, *p* < 0.0001; ∗∗∗, *p* < 0.001; ∗∗, *p* < 0.01.

### Genome targeting in neonate Fabry mice results in complete lyso-Gb3 clearance with the higher AAV dose

We then tested the strategy in neonate P5 Fabry mice, aiming at obtaining higher gene targeting rates and more efficient clearance of lyso-Gb3 in affected organs, with the potential to apply the treatment earlier in life in patients diagnosed by the neonatal screening programs. Since in preliminary experiments the treatment of neonate mice with fludarabine resulted in toxicity (not shown), we performed the treatment with only the donor vector containing the promoterless WT or the codon optimized h*GLA* cDNAs co-administered with AAV8 Cas9, at two doses: 3.0E13/vg/kg and 3.0E14 vg/kg (Figure 3A). Blood samples were taken at different timepoints, and mice were sacrificed at P150. No differences in the growth curve were observed (Figure S3A). α-Gal A enzyme activity and lyso-Gb3 levels in plasma were constant for the duration of the experiment (Figures 3B and 3C). For all treated groups and tissues analyzed, the α-Gal A enzyme activity was higher than that in the control WT group (Table S4 shows the values at P150), although the difference reached statistical significance in all tissues analyzed only for the high dose Donor CO02 + Cas9 group versus Control WT and Control KO groups, while the Donor WT + Cas9 versus Control WT and Control KO groups was significant only in plasma (Table S5). Lyso-Gb3 accumulation in plasma and tissues was significantly reduced for all treated groups, with the group treated with the CO02 *GLA* cDNA and Cas9, at the high dose, with levels similar to WT (Figures 3C and 3G–3K; Tables S4 and S5). In all cases, the mice treated with the CO02 *GLA* cDNA presented higher α-Gal A enzyme activity and lower lyso-Gb3 levels than the groups treated with the same dose of the WT *GLA* cDNA (Figures 3B–3K; Tables S4 and S5). It is important to note that as a comparison control, we performed weekly administrations of Replagal (1 mg/kg) to 3-month-old Gla ko mice (P90) for 2 months (sacrifice at P150), and both AAV-vector doses and cDNA versions cleared lyso-Gb3 more efficiently than weekly administrations of ERT (Figure S4).

**Figure 3:**
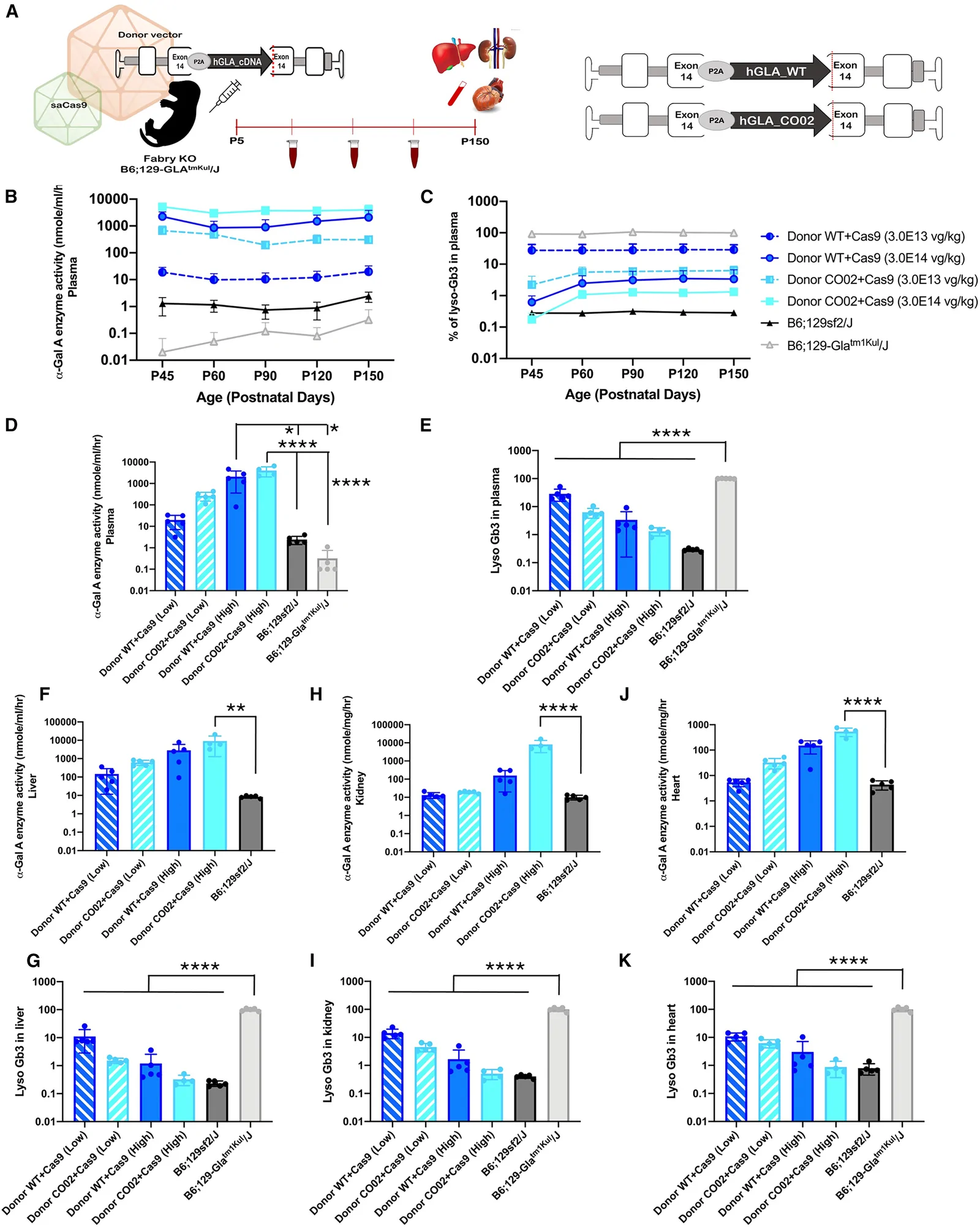
Genome targeting treatment of newborn P5 Fabry mice (A) Experimental scheme. Hemizygous male mice (B6;129-*Gla*^tm1Kul/J^; *n* = 5 per group) were treated with either AAV8-donor h*GLA* WT or AAV8-donorh*GLA* CO02, and the AAV-SaCas9 viral vectors at the dose of 3.0E13 vg/kg or 3.0E14 vg/kg. Animals were injected retro-orbitally at P5 and sacrificed at 5 months of age. Blood was collected at intermediate time points and at termination along with harvesting the liver, kidneys, and heart for analysis. Age-matched untreated hemizygous males (B6;129-*Gla*^tm1Kul/J^) and wild type (B6;129sf2/J) were used as controls; (B) α-Gal A enzyme activity was measured in blood plasma collected at different time points at P45, P60, P90, P120, and P150 for all treated and untreated animals. The graph shows the mean of the activity (nmoles/mL/h) at each time point. Mixed effects model (REML) *p* < 0.05; interaction, *p* < 0.0001; time, *p* < 0.0001; treatment, *p* < 0.0001. (C) Lyso-Gb3 levels were measured in blood plasma collected at different time points during the experiment. The lyso-Gb3 levels is indicated as a percentage, considering the mean lyso-Gb3 levels in untreated hemizygous mice as 100%. (D, F, H, and J) Bar graph representation of α-Gal A enzyme activity at P150 in plasma (D), liver (F), kidney (H), and heart (J). Each dot represents a single animal. (E, G, I, and K) Bar graph representation of the relative lyso-Gb3 levels at P150 in plasma (E), liver (G), kidney (I), and heart (K). The lyso-Gb3 levels are indicated as a percentage, considering the mean lyso-Gb3 levels in untreated hemizygous mice as 100%. Each dot represents a single animal. One-way ANOVA (D, E, G, H–J, and K), ∗∗∗∗, *p* < 0.0001; (F) ∗∗, *p* = 0.0046.

These results were confirmed by the western blot analysis of liver protein extracts (Figures S3B and S3C). The genome targeting rate presented no difference between the two AAV donor vectors, for each dose (Figure S3D; Table S6).

### Co-administration of AAV-donor vector with AAV-SaCas9 results in higher therapeutic efficacy than co-treatment with fludarabine

Next, to assess the effect of the fludarabine treatment in neonate mice, we tested different administration protocols of the drug using a GFP reporter donor construct (Figure S5A), aiming at identifying milder, less toxic conditions of the treatment. The only treatment showing a clear increase, compared with the “eGFP Donor DNA” group (no fludarabine), involved the shortening of the treatment to 2 days, with half-dose injected twice during each day (Figures S5B–S5D). In view of the better performance in newborn mice of the codon-optimized *GLA* cDNA, compared with the WT version, the next experiments were performed only with the CO cDNA donor construct. Thus, we treated neonatal P5 Fabry mice with the donor construct containing the promoterless codon-optimized h*GLA* cDNA alone (3.0E14 vg/kg) or co-administered with AAV8 Cas9 or fludarabine treatment. The treatment with SaCas9 resulted in the highest α-Gal A enzyme activity in plasma, liver, heart, and kidney, although in kidney the difference did not reach statistical significance (Figures 4A–4C; Tables S7 and S8). When compared with the donor-only treated group, the treatment with fludarabine resulted in a minor, non-statistically significant, improvement in enzyme activity in plasma, and similar levels in liver, kidney, and heart (Tables S7 and S8). In line with these results, the levels of lyso-Gb3 of the group treated with donor vector and SaCas9 presented the lowest levels in plasma, liver, kidney, and heart (Figures 4D–4H; Tables S7 and S8), which were similar to those of WT mice. The levels of lyso-Gb3 in all groups were lower than those of the untreated mutant mice.

**Figure 4:**
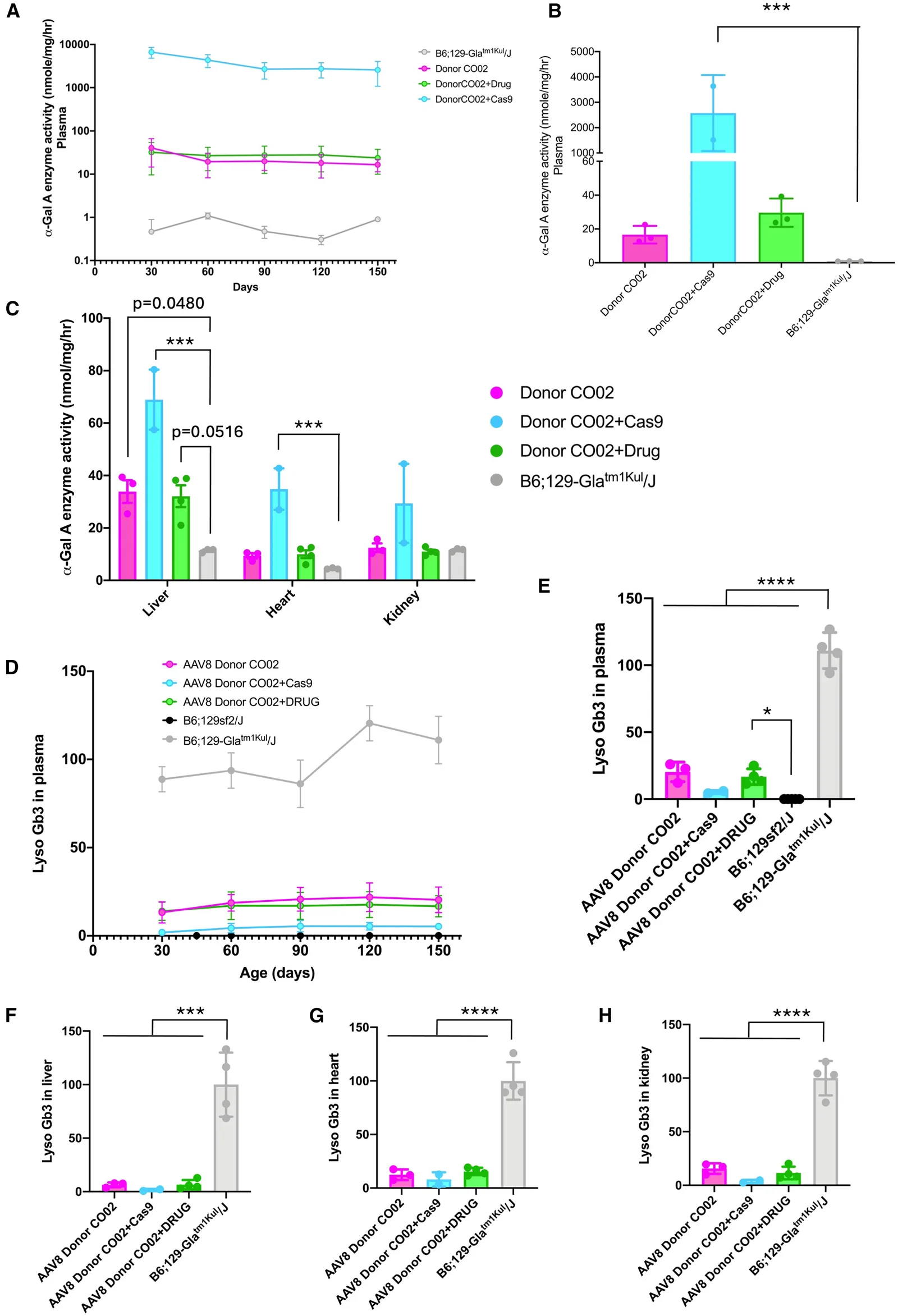
Genome targeting treatment of newborn P5 Fabry mice; comparison fludarabine vs. nuclease (A) α-Gal A enzyme activity was measured in blood plasma collected at different time points at P30, P60, P90, P120, and P150 for all treated and untreated animals. The graph shows the mean of the activity (nmoles/mL/h) at each time point. Two-way RM ANOVA: time × treatment: *p* < 0.0001; time: *p* < 0.0001; treatment: *p* < 0.0001. (B) Bar graph representation of α-Gal A enzyme activity at P150 in plasma. One-way ANOVA, *p* < 0.0047. (C) Bar graph representation of α-Gal A enzyme activity at P150 in liver, heart, and kidney. Each dot represents a single animal. One-way ANOVA, liver: ∗∗∗, *p* < 0.0006; heart: ∗∗∗, *p* < 0.0004; kidney: *p* = 0.098 NS. (D) Relative lyso-Gb3 levels were measured in blood plasma collected at different time points during the experiment. The levels present in Fabry mice correspond to 100%. (E–H) Bar graph representation of lyso-Gb3 levels at P150 in plasma (E), liver (F), heart (G), and kidney (H). Each dot represents a single animal. One-way ANOVA (E–H), *p* < 0.0001. Multiple comparisons, ∗, *p* < 0.05; ∗∗∗, *p* < 0.001; ∗∗∗∗, *p* < 0.0001.

These results showed that all treatments resulted in an important reduction of lyso-Gb3 levels in plasma and target organs (ranging from ∼70% to 99% reduction), with the treatment with SaCas9 being the most effective one.

### Hyposensitivity to heat is rescued in treated Fabry mice

The functional efficacy of the therapy was evaluated by challenging the treated mice with a test designed to assess hyposensitivity to heat.^32^^,^^56^^,^^57^ Thus, the neuropathy in Fabry mice treated with AAV-treated and control animals of Figure 4 was assessed in the hot plate test. We observed that the latency of untreated Fabry mice was 2- to 3-fold higher than WT control mice, and significantly higher than all Fabry mice treated with AAV gene targeting vectors (with and without Cas9, or with fludarabine) (Figure 5; Table S9). On the contrary, Fabry mice treated with AAV gene targeting vectors (with and without Cas9) behaved as WT untreated healthy animals and latency time was significantly lower than control 129-Gla^tm1Kul/J^ mice (Figure 5; Table S9), indicating that the treatment of presymptomatic Fabry mice can prevent the decline in peripheral sensory function, although the mice treated with AAV-donor+fludarabine did not reach the WT score (Table S9: one-way ANOVA *p* < 0.001; multiple comparisons: Mutant vs. all treated groups, *p* < 0.01; B6 vs. drug, *p* = 0.02; B6 vs. only donor, *p* = NS; B6 vs. Donor + Cas9, *p* = NS). These results strongly indicate that targeted integration of the *GLA* cDNA into the albumin locus of Fabry mice (enhanced by Cas9 co-administration) was able to correct not only the biochemical deficits but also a measurable functional neuropathy.

**Figure 5:**
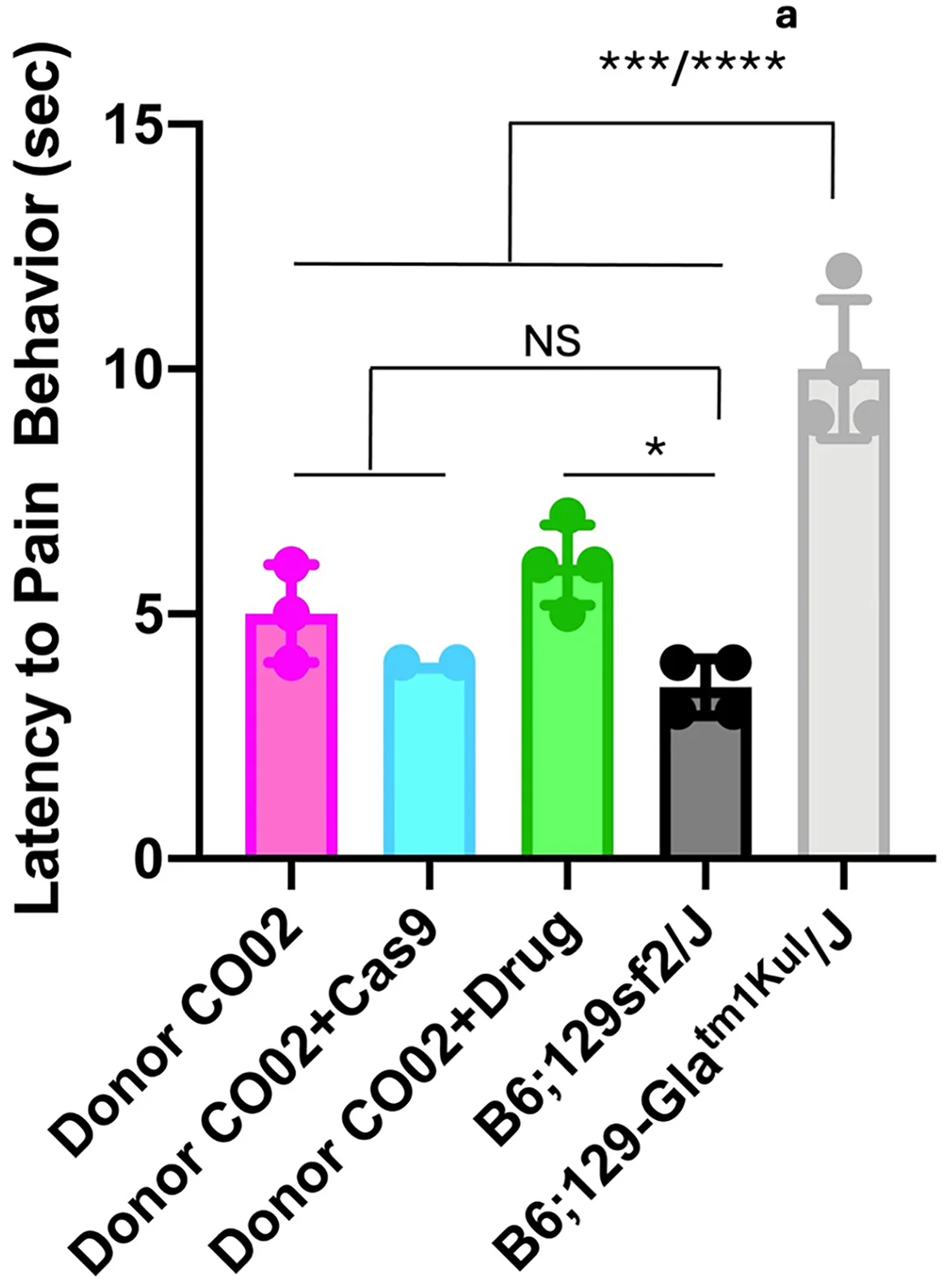
Functional evaluation of Fabry KO mice treated the genome targeting strategy at P5 Hot plate test. Treated and untreated control mice were challenged with the hot-plate test and latency to pain was recorded. Each dot indicates a single animal. One-way ANOVA, *p* < 0.001; Tukey’s multiple comparisons, ^a^*Gla*-ko vs. Donor and Donor + Drug, *p* < 0.001; ^a^Gla-ko vs. Donor + Cas9 and WT, *p* < 0.0001. ∗∗∗*p* < 0.001; ∗∗∗∗*p* < 0.0001.

### Genome targeting in human cells requires high sequence homology

To test the potentiality of the approach in a human-like context, we generated two donor constructs containing the human albumin homology arms with the two most common haplotypes found in the population (Hap1 and Hap2) flanking the P2A and the eGFP reporter gene (Figure S6A). These vectors were encapsidated into the LK03 AAV serotype, which infects human hepatocytes more efficiently than the AAV8 serotype, and infects mouse hepatocytes with much lower efficiency.^58^ We first set up a TaqMan-Real time PCR assay to verify the haplotype of Huh-7 cells and of human blood samples. The results showed that the Huh-7 cell line was homozygous for the haplotype 2, with one human sample homozygous for haplotype 1 and three heterozygous (Figure S6B). Next, we infected Huh-7 cells with both AAV-human donor vectors (Hap1 and Hap2), with or without co-infection with the LK03 AAV serotype coding for the SaCas9 and sgRNA (Figure 6A) and assessed the levels of the chimeric albumin-eGFP mRNA by RT-qPCR (Figure 6B). The only PCR product possible results from the chimeric albumin-eGFP mRNA, since the forward primer anneals outside the region of homology and the reverse primer anneals in the eGFP coding sequence. As expected, the expression of the chimeric mRNA resulting from the targeting of the constructs differed substantially between the two haplotypes. The mRNA expression of the cells infected with the Donor-Hap2 targeting vector was higher than those infected with the Donor-Hap1 (Figure 6B). We hypothesize that this difference could be related to the increased homologous recombination rate when isogenic DNA is employed,^59^^,^^60^ since the Huh-7 cells have the haplotype 2 (Figure S6B). We also observed that the cells co-infected with the AAV vector expressing the SaCas9 presented higher expression of the chimeric mRNA compared with the cells treated with the donor AAV alone. Finally, we tested the approach by infecting a primary culture of human hepatocytes (Figure 6C). We observed that the mRNA expression of the cells infected with the Donor-Hap2 targeting vector was roughly 100× higher than for cells infected with the Donor-Hap1. This result was justified by the fact that the analysis of the human primary hepatocytes revealed their haplotype was Hap-2 (Figure 6D).

**Figure 6:**
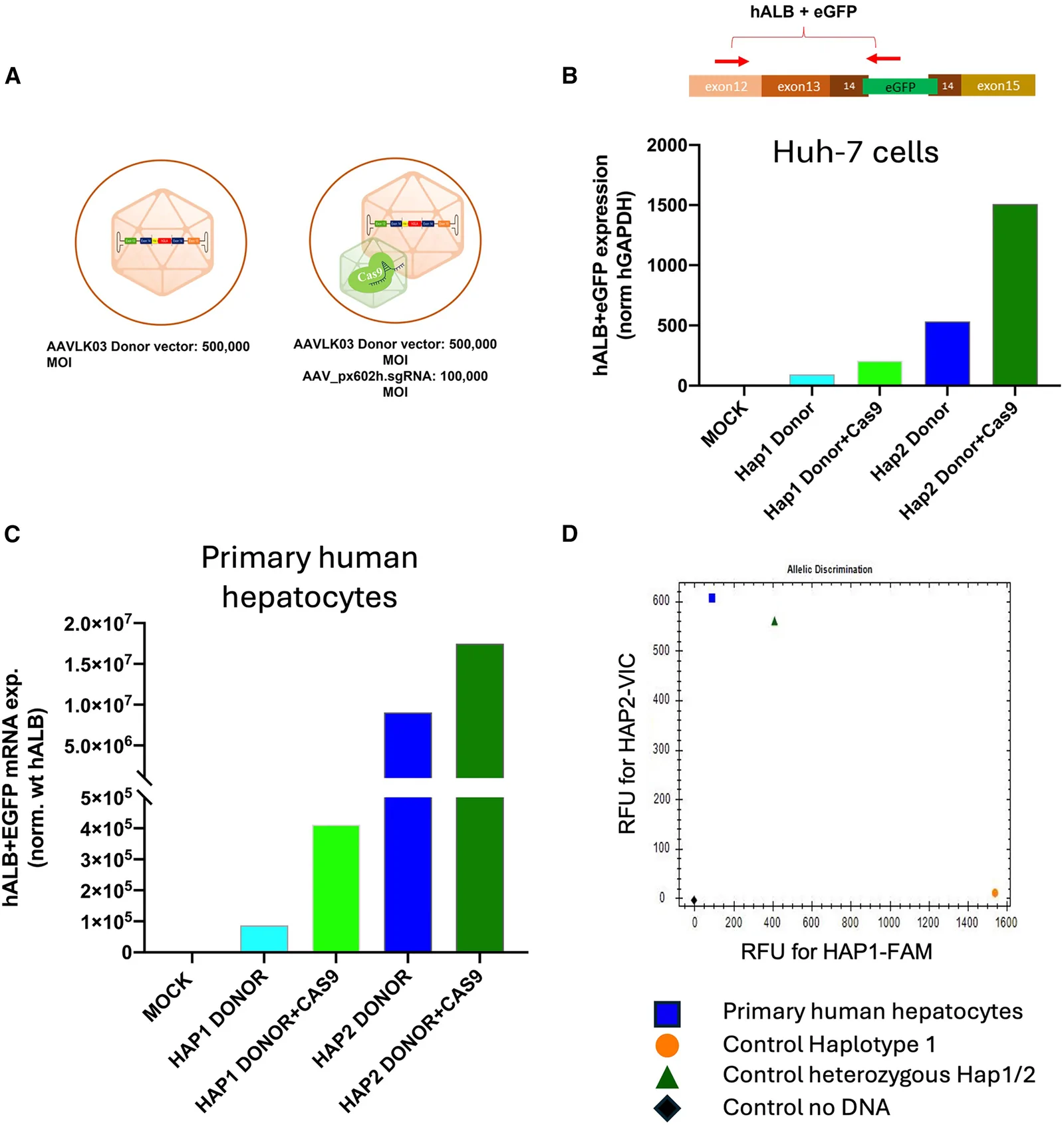
Genome targeting in a human liver cell line and in primary culture of human hepatocytes (A) Scheme of the two treatments: AAV-LK03 donor vector alone or with AAV-LK03 SaCas9 vector. (B) qRT-PCR of total mRNA from Huh-7 liver cells infected with the AAV vectors. The scheme of the PCR is indicated on the top of the image. (C) qRT-PCR of total mRNA from primary culture of human hepatocytes infected with the AAV vectors. (D) Taq-Man PCR of genomic DNA from a primary culture of human hepatocytes and human blood samples used as control.

These results strongly support the potentiality to translate the genome targeting approach to the clinic and confirm the requirement of full homology between the donor vector and the target gene to achieve higher genome targeting rates.

## Discussion

In most countries, classical and late-onset FD is diagnosed in childhood and adulthood, respectively. However, the implementation of newborn screening will facilitate earlier and faster diagnosis, leading to the possibility of initiating the therapeutic intervention much earlier than the onset of the first symptoms. Newborn screening is being employed in different countries^20^^,^^21^ and, as it becomes more widely implemented across countries, there will be an increasing medical need for developing more effective therapeutic approaches for the early treatment of FD. Moreover, the development of therapeutic strategies effective in newborns and children affected with FD is particularly important since the treatment is more efficient if initiated early, before the onset of symptoms and irreversible organ damage.^61^

ERT, the current standard of care for the treatment of patients with FD, is a non-curative therapy and has many limitations and elevated costs. AAV-based liver-directed episomal gene therapy is an interesting alternative approach in which the hepatocyte-produced α-Gal A enzyme is secreted into the circulation and captured by tissues and organs expressing mannose-6-phosphate receptors, as it occurs with ERT. This strategy has been tested in pre-clinical models of FD and in clinical trials for adult patients with FD.^26^^,^^27^^,^^28^^,^^30^^,^^62^^,^^63^^,^^64^^,^^65^^,^^66^^,^^67^^,^^68^^,^^69^^,^^70^ However, this approach, with proved safety and efficacy for the treatment of adult patients suffering from monogenic disorders,^29^^,^^33^^,^^43^^,^^71^^,^^72^^,^^73^ presents important limitations in neonatal and pediatric settings, as the viral episomal DNA^74^ may be lost in the growing liver.^39^^,^^40^^,^^41^ The loss of vector DNA may also gradually occur in the homeostatic healthy liver (and in conditions associated with liver damage), as human hepatocytes show continuous and lifelong turnover, with Ki67 labeling index <0.5%^75^ and 17% hepatocyte turnover per year in adults.^76^ Consequently, the therapeutic efficacy is also reduced over time requiring, very probably, re-administration of the therapeutic vector,^77^ not yet possible due to the presence of neutralizing antibodies against the AAV capsid generated during the first vector administration.^33^^,^^42^^,^^43^ Vector loss in both pediatric and adult settings is still a major challenge of AAV-based episomal gene therapy and different approaches to solve this limitation are under experimentation.^78^^,^^79^^,^^80^^,^^81^^,^^82^^,^^83^^,^^84^^,^^85^

Genome targeting has the potentiality to be more effective than both ERT and AAV-based liver-directed episomal gene therapy. It overcomes the loss of AAV episomal vectors in the growing liver and in conditions resulting in hepatocyte proliferation as the hepatocyte genome is permanently modified with the therapeutic gene, which is then transmitted to daughter cells upon cell duplication, with long-term therapeutic benefits.^25^ One of the main advantages of genome targeting over ERT resides in the one-time treatment, avoiding life-long and costly bi-weekly infusions required for ERT,^8^ with a significant improvement not only in the correction of disease symptoms but also in the patient life quality. Additionally, it enables continuous production of the therapeutic enzyme, resulting in constant levels of recombinant α-Gal A activity, unlike the fluctuating enzyme levels associated with ERT due to its short half-life in circulation^11^^,^^12^^,^^13^ limiting, at the same time, the risk of formation of ADA^26^^,^^27^ due to the tolerogenic properties of the liver.^34^^,^^35^

Since the expression of the α-Gal A provides no proliferative advantage to hepatocytes, amplification of the targeted hepatocytes is not expected to occur in our model, as opposed to genome targeting the methylmalonyl-CoA mutase cDNA in a mouse model of methyl malonic acidemia (MMA), which presents amplification of the edited hepatocytes.^86^ This approach significantly differs from ours, as no nuclease was used to obtain higher genome targeting rates, while we used a nuclease, resulting in at least ∼8% of targeted hepatocytes. Importantly, in the MMA model, there is no cross-correction of non-edited cells/tissues, in contrast with our strategy in which the non-corrected cells in affected tissues benefit from the high levels of circulating α-Gal A enzyme produced and secreted by the targeted hepatocytes by capturing the enzyme and correcting the disease phenotype.

Recently, programmable base-editor nucleases were used to correct a mutation in babies suffering from neonatal-onset carbamoyl-phosphate synthetase 1 (CPS1) deficiency, an ultra-rare inborn error of metabolism affecting the urea cycle.^87^ FD mutation-specific approaches using CRISPR-Cas9 and base editors have been tested in iPS cells and cell lines,^45^^,^^88^ but not yet in animal models. While these CRISPR-Cas9-mediated and base editor approaches are personalized, as each single mutation requires the development and testing of mutation-specific reagents, our approach is intended to correct most of the disease-causing mutations in the *GLA* gene.

In the present study, we tested AAV-mediated genome targeting gene therapy for two disease-relevant ages using a widely recognized mouse model of FD^89^: newborn mice, to verify the efficacy of a therapeutic approach that might be relevant to treat pre-symptomatic patients diagnosed by newborn screening programs with the classic form, and juvenile mice, at 1 month of age, to validate its use later in life in late-onset and less severe forms of FD. The genome targeting strategy used here is based on the GeneRide approach developed at Mark Kay’s lab^47^ and tested in our lab in newborn animals of a model of Crigler-Najjar syndrome I,^50^ later modified to increase gene targeting rate by the use of SaCas9^48^ or fludarabine.^49^ Fludarabine was tested in WT and in hemophilia B mice, showing 3- to 6-fold increase in targeting rate compared with control mice.^49^ We have also successfully tested the GeneRide-Cas9 combined approach in citrullinemia type I and hemophilia B mice, obtaining improved gene-targeting rate compared with spontaneous homologous recombination (no nuclease or drug).^51^^,^^52^ The use of SaCas9 to increase gene-targeting rate results in high-precision targeting of the desired transgene into the albumin locus, with undetected off-target activity in predicted sites or other undesired secondary effects, such as variations in albumin levels or histological alterations.^48^ In fact, using the same tools applied here, we had previously tested both on- and off-target activities of the SaCas9 nuclease without observing off-target activity above background levels in predicted sites. There were no predicted off-targets having less than four nucleotide mismatches, and only four potential off-target sites were identified with four mismatches.^48^ This could be associated with the more complex PAM site (NNGRRT), compared with SpCas9 (PAM site: NGG).^90^ Thus, in this model, we also expect no significant off-target activity, although additional safety studies may be required, such as determination of precise donor integration and a more detailed evaluation of potential off-target sites and potential secondary effects to safely transfer this approach to the clinic.

We showed that our approach resulted in supraphysiological levels of α-Gal A enzyme activity in plasma and target organs, indicating that the liver-produced enzyme was efficiently secreted into the circulation and subsequently taken up by tissues and organs. The efficacy of the approach was further supported by the complete or almost complete clearance of Lyso-Gb3 in affected organs of Fabry mice, with steady levels maintained throughout the duration of the experiment without loss of therapeutic efficacy, suggesting that the treatment did not produce evident cellular stress or other adverse effects, although more detailed safety studies may be required to rule out undesired long-term adverse effects. Furthermore, the sustained efficacy suggests that the recombinant hepatocytes remained stable over time and that the treatment did not induce significant levels of neutralizing antibodies against the liver-derived human enzyme. Our results are in line with previous reports showing the induction of immune tolerance after AAV-mediated hepatic expression of α-Gal A^27^ and other therapeutic proteins,^35^^,^^91^^,^^92^ although direct experimental validation of this assumption is still required.

The animals treated at P30 with fludarabine, and the donor *GLA* cDNA presented the lower lyso-Gb3 levels in target organs, reaching 90% reduction in heart, 90% in kidney, 95% in liver, and 85% in plasma. No major differences were observed between the WT and the codon-optimized *GLA* cDNA versions in the P30-treated mice. Unexpectedly, in the P30-treated animals, we observed a low correlation between α-Gla A activity and lyso-Gb3 levels. Indeed, the SaCas9-treated groups showed the highest α-Gal A activity in plasma and tissues when protein extracts were tested *in vitro*. However, this was not associated with a better clearance of lyso-Gb3 in these tissues, compared with the other treated groups (no SaCas9 and fludarabine-treated groups). One possible explanation may involve the presence of different hepatocyte populations in the P30 mice associated with the establishment of metabolic zonation after weaning,^93^^,^^94^^,^^95^ with subtle differences in the recombination mechanisms and genome targeting rate (spontaneous recombination in the absence of nucleases, DSB-induced HDR, or HDR-mediated by fludarabine-induced DNA damage). These different hepatocyte populations could also present differences in protein folding or in post-translational modifications of α-Gal A, affecting the correct intracellular trafficking and localization following enzyme uptake by the target tissues. Such alterations may not be detected by the *in vitro* assay, which measures enzyme activity in whole-tissue extracts, but could result in reduced functional activity *in vivo*. In addition, the proposed post-translational modifications or folding differences could lead to a different activity toward the natural substrate that may not be captured by the *in vitro* assay using an artificial one. However, these hypotheses remain speculative and require thorough experimental validation.

On the contrary, treatment at P5 with Donor plus Cas9 was very effective, favored by the active HDR mechanisms in dividing hepatocytes,^95^ reaching complete lyso-Gb3 clearance in plasma, and target tissues with the high dose, and almost complete clearance with the low dose (heart, 94%; kidney, 96%; liver, 99%; and plasma, 94%), with a good correlation between enzyme activity and lyso-Gb3 levels. In the newborn scenario, the CO02 *GLA* cDNA was significantly more efficient than the WT *GLA* cDNA version (Figure 3; Tables S4 and S5) although, unexpectedly, this difference was not evident in some of the juvenile-treated groups (Figure 2 and Table S1). A study by Pagant et al.^96^ tested a genome-targeting approach in Fabry mice using zinc finger nucleases targeting the first intron of the albumin gene to introduce and express the *GLA* gene in hepatocytes. In this study, 2-month-old mice were dosed with 1.20E12 vg/mouse or 4.0E11 vg/mouse (corresponding to approx. 6.0E13 vg/kg and 2.0E13 vg/kg, respectively) resulting, with the low dose, in 7% of residual lyso-Gb3 in plasma and liver, and 10% in kidney.^96^ While the doses and results are comparable with the ones applied here in the mice dosed at P30, the integration mechanism is different, as the authors report that most integration events occurred by NHEJ, rather than HDR. In our approach, the donor vector integrates through a homology-directed repair mechanisms.^47^^,^^49^ The integration rate in juvenile P30-dosed Fabry mice was about 1% (Figure S2C), in line with the HDR rate obtained in adult animals by Pagant et al.^96^ and with the lower proliferation activity of hepatocytes. In contrast, treatment of mice at P5, a phase in which hepatocytes are still in active proliferation and the HDR mechanism is highly active,^95^ the gene targeting rate reached 8%–9% with the high vector dose (Figure S3D; Table S6). In the treatment of P30 animals, despite the high α-Gal A enzyme activity observed in the group of mice receiving AAV-donor CO02 and SaCas9, the levels of lyso-Gb3 were, unexpectedly, higher than in the other groups (Figure 2), although the difference was not statistically significant.

To summarize, these results indicate that a near-complete reduction of lyso-Gb3 accumulation was achieved with 1%–2% of hepatocytes permanently modified to express the human α-Gal A enzyme. The efficacy of the treatment is also supported by the normalization of the neurological phenotype, as assessed by the hot plate test in treated Fabry mice, strongly suggesting that treatment of presymptomatic patients of FD may prevent the decline in peripheral sensory function. The genome-editing strategy described here leads to the permanent integration of the h*GLA* transgene and demonstrates efficacy in FD mice treated during the neonatal and juvenile age, supporting the potential clinical translation of the approach for the treatment of both children and/or adolescents with classical or late-onset forms of FD, respectively.

## Materials and methods

### Animals

All animals were housed and handled according to the institutional guidelines and the Italian Ministry of Health in the ICGEB bio-experimental facility in Trieste (Project Authorization N. 365/2021-PR), following European Union (EU) Directive 2010/63/EU for animal experimentation and ARRIVE guidelines. Mice were maintained in a temperature-controlled environment with 12/12 h of light/dark cycles and received a standard chow diet and water *ad libitum.* B6;129-*Gla*^tm1Kul/J^ (*Gla* ko mice, Strain #003535) were purchased from Jackson laboratory, and the colony was amplified in the ICGEB bio-experimental facility. Only male mice were used for experimentation.

### Animal procedures

AAV viral vector treatment was done intravenously via the retro-orbital route, dosed as AAV viral genomes per animal weight (vg/kg) in isoflurane anesthetized animals as previously described.^97^

Fludarabine treatment was done in WT or Fabry KO by treating P5 or P30 male mice intraperitoneally. For P30 mice, the drug was administered at 125 mg/kg (total 375 mg/kg/day) three times a day with an interval of 3–4 h for three consecutive days. This treatment was coupled with a single AAV retro-orbital injection on day 1 preferably between the first and second dose. For the P5 mice, to reduce toxicity, the administration of the dose was reduced to 190 mg/kg/day in three of the tested groups (the fourth one received 380 mg/kg/day), for two days with the dosing schedule indicated in Figure S5A.

For ERT treatment, 3-month-old *Gla* KO male animals were treated with 1 mg/kg agalsidase alfa (Replagal Takeda, ERT) every week for 2 months. Blood samples were taken every 2–4 weeks, and mice were sacrificed at P150, 2 months after the first ERT dosing.

To assess pain sensitivity, a hot plate assay was conducted on age-matched male mice as previously described with minor modifications.^26^ Following a 20-min acclimation period, each mouse was placed on a 50 °C heated metal surface enclosed by an 18-cm diameter cardboard wall to prevent escape. A timer was started, and the latency (in seconds) to the first sign of nociception (hind paw licking, shaking, flicking, jumping, or vocalizing) was recorded. A 30-s cut-off was implemented to avoid tissue damage.

### rAAV vector production

The AAV vectors used in this study are based on AAV type 2 backbone, and infectious vectors were prepared by the AAV Vector Unit at ICGEB Trieste, in HEK293 cells by a cross-packing approach whereby the vectors were packaged into AAV capsid 8 (dosed to mice) or in LK03 (dosed to human hepatocytes) serotypes, as described previously.^98^

### Cell experiments

Huh-7 cells were maintained in Dulbecco’s Modified Eagle Medium (DMEM) with 10% of Fetal Bovine Serum (FBS) and antibiotics at 37 °C. For the experiment, Huh-7 cells were seeded in a 96-well plate on day 0 by triplicate. Cells were treated with Fludarabine on day 1 by replacing the growth medium with fresh DMEM containing 100 μM fludarabine. Drug-treated cells were incubated for 16–18 h. On day 2, transduction with AAV vectors was performed by diluting AAV donor vector at 500,000 MOI in OptiMEM. In the case of Donor vector+Cas9 treatment, AAVLK03px602hsgRNA and AAV donor vector were diluted (1:5) in OptiMEM together at 100,000 and 500,000 MOI, respectively, and added to the cells. The transduced cells and mock untreated cells were harvested for analysis.

The primary hepatocytes were acquired from Cytes Biotechnologies S.L., Barcelona, Spain (Lot CHM2225-HE-Z) already plated, were maintained in the medium provided by the manufacturers, and were transduced at 1,000,000 MOI.

### Total protein liver extraction and western blot analysis

Different lysis buffers were used depending on the analytical purposes. Lysis buffer for western blot analysis was composed of 50 mM HEPES (pH 7.4), 150 mM sodium chloride (NaCl), 1 mM EDTA, 0.5% NP40, and protease inhibitor. The buffer used to extract proteins to perform α-Gal A enzymatic assay consisted of 28 mM citric acid, 44 mM disodium phosphate, 1% Triton X-100, and protease inhibitor. Protein liver homogenates of treated and untreated mice (15 μg) or plasma (diluted 1:50) were separated in an SDS-PAGE, then transferred to a polyvinylidene fluoride (PVDF) membrane, and blocked in 5% milk in 0.1% PBS-Tween 20 for 2–4 h, followed by incubation with the primary antibody (rabbit anti-h-α-Gal A, Sino Biologicals, 1:3,000, cat: 12078-R001; rat anti-HSP70 Enzo Life Science, 1:3,000, cat ADI-SPA-815-D), and then with a goat anti-rabbit IgG horseradish peroxidase (IgG-HRP) or anti-rat, respectively (goat anti-rat IgG-RP, Bethyl Laboratories, 1:3,000, cat A110-305P; goat anti-rabbit immunoglobulins HRP, Dako, 1:3000, cat P0448). The membrane was developed with Enhanced Chemiluminescence (ECL – ThermoFisher Scientific). Band intensities on the images were measured using Image Lab software 6.0.1 (Biorad).

### Histology

The liver and kidney were harvested in 4% PFA (4% paraformaldehyde in PBS) and fixed in the solution at 4 °C for 24 h and maintained in 20% sucrose, 0.02% sodium azide, in PBS.

For immunofluorescence, the tissues were sliced into 4 μm slices on histological slides and subjected to antigen retrieval (10 mM sodium citrate buffer pH 6.0) by baking the slides in the microwave for 40 min. After washing with PBS and blocking (5% BSA in 0.4% PBS-Triton X 100), sections were incubated with the primary antibody in 2.5% BSA in 0.4% PBS-triton X 100 (mouse anti-Gb3, Amsbio, 1:250, cat AMS.A2506; Alexa Fluor 488 Phalloidin, Invitrogen, 1:500, act A12379), incubated with a secondary antibody (Alexa Fluor 568 goat anti-mouse IgG, Invitrogen, 1:500, cat A11004), counterstained with Hoechst and mounted in Mowiol. Slides were imaged with Leica fluorescent microscope (Leica DMIL LED) coupled with DFC450C camera. Between 15 and 20 slices per animal were analyzed and the mean calculated. Images were processed with ImageJ software.

### α-Gal A enzyme activity

α-Gal A enzyme activity test was done with Huh-7 cell extracts and supernatant medium, and murine plasma and tissue including liver, kidney, and heart as previously described with minor modifications.^53^^,^^99^^,^^100^ Different concentrations (2, 4, 10, 20, 40, 80, and 100 pmoles) of 4MU (4-Methylumbelliferone, Cat# M1381 Sigma Aldrich) were used as standard to generate a reference curve. In a black opaque 96-well plate, a 2 μL enzyme-containing sample (protein from cell/tissue, supernatant medium, or blood plasma) and 20 μL of 4MUG (4-methyl-umbelliferyl-alpha-D-galactopyranoside, Cat#M7633 Sigma Aldrich) (2.46 mM 4MUG in 0.1 M Citrate/0.2 M phosphate buffer pH 4.5) substrate were incubated at 37°C for 1 h. The reaction was stopped with 200 μL of 200 mM glycine-NaOH Buffer pH 10.4. The released fluorescence was measured at excitation 365 nm and emission 450 nm wavelengths with a multi-plate reader (PerkinElmer Envision Plate Reader, Waltham, MA). For the analysis, the RFU obtained was normalized with the appropriate blank samples, and 4MU units (B) released by the samples were calculated using the standard curve and expressed in nmoles/mL/h or nmoles/mg/h as indicated in the figures.

### Lyso-Gb3 quantification

Lyso-Gb3 level was evaluated in murine plasma and tissues (liver, kidney, and heart) of treated and untreated animals. For tissues, 20 mg of powdered tissue was homogenized using a mechanical homogenizer (IKA Ultra-Turrax T25) with 350 μL of water. Tissue and plasma lyso-Gb3 concentrations were determined by liquid chromatography-mass spectrometry (LCMS). Homogenized samples or plasma were mixed with precipitation solution (10 mL internal standard solution [1–10 ng/mL], 10 mL acetone, 2.22 mL water) containing internal standard (1 ng/mL lyso GlcCer d5 - lyso-SM d9; 10 ng/mL lyso-Gb3 d7 in methanol). The mix was vortexed and incubated on an orbital shaker for 30 min and then centrifuged at 16,000 rcf for 10 min. The clear supernatant was collected and dried under a nitrogen stream. The dried content was reconstituted with 100 μL mobile phase (55% FMA: water + 0.1% HCOOH, 45% FMB: CH_3_CN + 0.1 %HCOOH) and centrifuged at 16,000 rcf for 5 min 100 μL supernatant was used for analysis by injecting 2 μL in Citrine Sciex 6500QT with Poroshell 120 EC-C8 2.7 μm and 3.0 × 50 mm columns. The analysis was done against a calibration curve prepared by spiking the pure standards in water: methanol 50:50 solution (range 0.1–200 ng/mL for lyso-Gb3, lyso GlcCer, and range 1–2,000 ng/mL for lyso SM/509).

In some figures, the absolute values of lyso-Gb3 accumulation in plasma (ng/mL) and tissues (ng/mg) were converted to percentages for ease of understanding. In the case of plasma, the mean of lyso-Gb3 accumulation in untreated hemizygous males (*n* = 5) at P150 was considered as 100%. Whereas, in the case of tissues, the mean of lyso-Gb3 accumulation in the specific organ (liver/kidney/heart) of untreated hemizygous males (*n* = 5) was considered as 100% accumulation. All the treated and untreated WT substrate levels were re-calculated as percentages based on this criterion.

### RNA preparation and RT-PCR

RNA was isolated using a NucleoZOL solution from Macherey-Nagel, following the manufacturer’s instructions, as previously described.^53^ For the RT reaction, 1 μg of total RNA was reverse-transcribed using M-MLV reverse transcriptase (Invitrogen, Carlsbad, CA, United States) following the manufacturer’s instructions, as previously described.^53^

### Genomic DNA preparation from cells and from liver samples

Genomic DNA was prepared from cells as briefly described below. Cells were lysed in 50 mM Tris-HCl pH 7.5, 100 mM EDTA, 0.5% SDS, and 200 μg/mL of protease K for 1 h at 37°C and centrifuged for 10 min at maximum speed. Genomic DNA was precipitated with 2-propanol and resuspended in TE buffer after centrifugation and washing.

Whole livers were extracted and reduced to powder with a mortar and liquid nitrogen, and stored at −80°C. Genomic DNA was prepared incubating as previously described.^101^

### SNP hALB genotyping

TaqMan predesigned SNP genotyping assay from Thermofisher Scientific was used to genotype the SNP ID rs962004, using fluorophores for human ALB Haplotype 1 (FAM/TTGTTGAGCTT) and Haplotype 2 (VIC/TTGTTGAGCTC). The sequence of the primers used for the genomic amplification was not provided by the company. Genomic DNA (20 ng) isolated from untreated Huh-7, and four different human blood samples were used to increase confidence in the genotyping assay. Water was used as the negative control. A reaction mix containing 2× iQ supermix (Biorad), 20× SNP assay mix, and water. DNA was added to the mix and run on a C1000 Thermal Cycler CFX96 Real-Time System (Bio-Rad). PCR conditions were as follows: 10 min at 95°C, plus 40 cycles of 15 s at 95°C and 1 min at 60°C. The hGAPDH was used as reference gene for normalization. The relative fold change was calculated using the Delta-Delta-Ct method. The generated dot plot was generated according to the Fluorophore-specificity in the sample.

The human blood samples were obtained from the members of the Mouse Molecular Genetics group (ICGEB, Trieste) who volunteered, and consent was not required.

### ddPCR

The on-target recombination rate was measured using ddPCR of liver genomic DNA as described previously.^49^ Briefly, 100 or 200 ng of gDNA was digested with SpeI for 1 h. 25 ng of digested gDNA was added to a 25 μL PCR reaction containing ddPCR Supermix for Probes (No dUTP) (Bio-Rad), 900 nM target-specific primers, and 250 nM amplicon-specific probes. Droplets were generated using 22 μL of PCR reactions and 70 μL of oil according to manufacturer’s instructions. Reactions were cycled as follows: 95°C for 10 min, 50 cycles of 95°C for 30 s, 60°C for 30 s, and 72°C for 6 min, and one cycle of 95°C for 10 min and held at 4°C until droplet reading. Primers to amplify a 1.6 kb non-targeted region of endogenous mouse Albumin were (5′-CTGCTGTGCACCAGTTGATGTT-3′) and (5′- TGCTTTCTGGGTGTAGCGAACT-3′), combined with a HEX-labeled probe (5′- TCTGGTGCTGAGGACACGTAGCCCAGT -3′). Primers to amplify on-target HR with a 1.4 kb amplicon were (5′-GGGCAAGGCAACGTCATGG-3′) and (5′- CCAGGGTTCTCTTCCACGTC-3′), combined with an FAM-labeled probe (5′-GCCCAAGGCTAC AGCGGAGC-3′).

### Statistics

Statistical analyses were performed for all the assays using GraphPad Prism 8.2.1. Data in the graphs were plotted as mean with the corresponding standard deviation (SD). Statistical analysis of data containing different time points was done using the mixed effects model (REML). The comparison of groups was done using one-way, two-way ANOVA, or mixed-effects model (REML), with post-hoc tests for statistical comparisons (Tukey’s multiple comparisons test). A *p* value below 0.05 was considered significant (∗*p* < 0.05, ∗∗*p* < 0.01, ∗∗∗*p* < 0.001, ∗∗∗∗, *p* < 0.0001).

## Data and code availability

The materials presented here are fully available for distribution after the execution of a material transfer agreement (MTA) with the respective institutions.

## Acknowledgments

We would like to thank the staff of the bioexperimentation facilities for technical support. This work was supported by the 10.13039/501100002426Telethon Foundation (grant no. GGP20128, to A.F.M.), Italy.

## Author contributions

H.S., A.D., and A.F.M. contributed to the experimental design, supervised experiments, and results interpretation, and wrote the manuscript. R.D., G.R., J.B., M.F., D.F., A.V., A.C., A.I., G.B., and L.Z. contributed to experimental design, performed experiments and analyzed the data, and contributed to results interpretation and manuscript preparation. H.S., G.R., G.B., A.V., and A.I. contributed to the *in vivo* experiments and experimental activities. All authors contributed to the revision and correction of the manuscript.

## Declaration of interests

A.F.M. and H.S. are owners of a patent describing the codon-optimized cDNA sequence for human alpha galactosidase (PCT/EP2024/055793).

## Supplemental information

Document S1. Figures S1–S6 and Tables S1–S9

Document S2. Article plus supplemental information
