# Supplementary material for: AAV-mediated genome targeting of the GLA cDNA in newborn and juvenile Gla KO mice achieves long-term correction of Fabry disease
Source: Mol Ther Adv. 2026 Oct 15;34(4):201864. doi: 10.1016/j.omta.2026.201864 (PMC13756261; doi:10.1016/j.omta.2026.201864)
Supplement: Supplementary file 1 [file mmc1.pdf]

OMTA, Volume *34*

Supplemental information

AAV-mediated genome targeting of the *GLA* cDNA in newborn and juvenile *Gla* KO mice achieves long-term correction of Fabry disease

Himanshi Saxena, Giulia Romano, Rossana Domenis, Jessica Biasizzo, Martina Ferro, Dania Ferino, Antonio Vicidomini, Andrea Colliva, Alessandra Iaconcig, Giulia Bortolussi, Lorena Zentilin, Andrea Dardis, and Andrés F. Muro

Supplemental tables

Table S1. α-Gal A enzyme activity and Lyso Gb3 (relative values) levels at P150 of the animals treated in Figure 2.

P150 Figure 2 (P30)	α-Gal A enzyme activity [Mean (SD)]				Lyso Gb3 [relative values; Mean (SD)]			
**Descriptive statistics**	**Plasma**	**Liver**	**Kidney**	**Heart**	**Plasma**	**Liver**	**Kidney**	**Heart**
**Donor WT**	6.27 (5.01)	12.36 (7.42)	16.17 (2.57)	3.62 (1.39)	34.11 (18.55)	16.34 (12.59)	24.38 (8.17)	26.74 (16.51)
**Donor CO02**	11.68 (3.95)	38.02 (21.49)	24.47 (5.22)	7.08 (2.32)	25.00 (4.00)	10.09 (3.34)	20.68 (4.57)	15.68 (5.18)
**Donor WT+Cas9**	522.0 (48.97)	719.8 (69.44)	1942 (904.8)	714.2 (199.8)	28.51 (4.37)	13.82 (4.80)	32.29 (11.45)	33.17 (9.61)
**Donor CO02+Cas9**	624.0 (279.4)	549.2 (241.2)	1369 (286.2)	684.5 (156.0)	61.46 (41.58)	45.38 (35.71)	63.99 (35.75)	57.64 (34.68)
**Donor WT+Drug**	34.83 (22.22)	149.6 (76.77)	20.72 (3.31)	7.71 (1.30)	15.09 (4.08)	5.16 (1.22)	10.47 (4.16)	15.79 (7.60)
**Donor CO02+Drug**	16.87 (7.62)	71.08 (23.51)	20.29 (2.63)	6.15 (1.97)	18.72 (3.97)	6.03 (3.05)	14.29 (5.36)	10.94 (3.42)
**B6;129sf2/J**	2.42 (1.00)	8.39 (1.30)	10.00 (2.91)	4.42 (1.75)	0.29 (0.03)	0.23 (0.05)	0.40 (0.05)	0.80 (0.35)
**B6;129-Glatm1Kul/J**	0.32 (0.44)	ND	ND	ND	100.0 (1.59)	100.0 (11.01)	100.0 (16.07)	100.0 (17.66)

Table S2. Statistical analysis (2-Way ANOVA; Tukey's multiple comparisons test) relative to α-Gal A enzyme activity and Lyso Gb3 (relative values) levels at P150 of the animals treated in Figure 2 and data shown in Table S1.

P150 Figure 2 (P30)	α-Gal A enzyme activity				Lyso Gb3			
**Tukey's multiple comparisons test**	**Plasma**	**Liver**	**Kidney**	**Heart**	**Plasma**	**Liver**	**Kidney**	**Heart**
**Donor WT vs. Donor CO02**	NS	NS	NS	NS	NS	NS	NS	NS
**Donor WT vs. Donor WT+Cas9**	<0.0001	<0.0001	<0.0001	<0.0001	NS	NS	NS	NS
**Donor WT vs. Donor CO02+Cas9**	<0.0001	<0.0001	<0.0001	<0.0001	NS	NS	0.0059	NS
**Donor WT vs. Donor WT+Drug**	NS	NS	NS	NS	NS	NS	NS	NS
**Donor WT vs. Donor CO02+drug**	NS	NS	NS	NS	NS	NS	NS	NS
**Donor WT vs. B6;129sf2/J**	NS	NS	NS	NS	NS	NS	NS	NS
**Donor WT vs. B6;129-Glatm1Kul/J**	NS	NA	NA	NA	<0.0001	<0.0001	<0.0001	<0.0001
**Donor CO02 vs. Donor WT+Cas9**	<0.0001	<0.0001	<0.0001	<0.0001	NS	NS	NS	NS
**Donor CO02 vs. Donor CO02+Cas9**	<0.0001	<0.0001	<0.0001	<0.0001	0.0341	0.0123	0.0021	0.0056
**Donor CO02 vs. Donor WT+Drug**	NS	NS	NS	NS	NS	NS	NS	NS
**Donor CO02 vs. Donor CO02+drug**	NS	NS	NS	NS	NS	NS	NS	NS
**Donor CO02 vs. B6;129sf2/J**	NS	NS	NS	NS	NS	NS	NS	NS
**Donor CO02 vs. B6;129-Glatm1Kul/J**	NS	NA	NA	NA	<0.0001	<0.0001	<0.0001	<0.0001
**Donor WT+Cas9 vs. Donor CO02+Cas9**	NS	NS	NS	NS	NS	NS	NS	NS
**Donor WT+Cas9 vs. Donor WT+Drug**	<0.0001	<0.0001	<0.0001	<0.0001	NS	NS	NS	NS
**Donor WT+Cas9 vs. Donor CO02+drug**	<0.0001	<0.0001	<0.0001	<0.0001	NS	NS	NS	NS
**Donor WT+Cas9 vs. B6;129sf2/J**	<0.0001	<0.0001	<0.0001	<0.0001	NS	NS	NS	NS
**Donor WT+Cas9 vs. B6;129-Glatm1Kul/J**	<0.0001	NA	NA	NA	<0.0001	<0.0001	<0.0001	<0.0001
**Donor CO02+Cas9 vs. Donor WT+Drug**	<0.0001	<0.0001	<0.0001	<0.0001	0.0032	0.0030	0.0001	0.0058
**Donor CO02+Cas9 vs. Donor CO02+drug**	<0.0001	<0.0001	<0.0001	<0.0001	0.0079	0.0039	0.0003	0.0016
**Donor CO02+Cas9 vs. B6;129sf2/J**	<0.0001	<0.0001	<0.0001	<0.0001	<0.0001	0.0007	<0.0001	0.0001
**Donor CO02+Cas9 vs. B6;129-Glatm1Kul/J**	<0.0001	NA	NA	NA	0.0211	<0.0001	0.0151	0.0047
**Donor WT+Drug vs. Donor CO02+drug**	NS	NS	NS	NS	NS	NS	NS	NS
**Donor WT+Drug vs. B6;129sf2/J**	NS	NS	NS	NS	NS	NS	NS	NS
**Donor WT+Drug vs. B6;129-Glatm1Kul/J**	NS	NA	NA	NA	<0.0001	<0.0001	<0.0001	<0.0001
**Donor CO02+drug vs. B6;129sf2/J**	NS	NS	NS	NS	NS	NS	NS	NS
**Donor CO02+drug vs. B6;129-Glatm1Kul/J**	NS	NA	NA	NA	<0.0001	<0.0001	<0.0001	<0.0001
**B6;129sf2/J vs. B6;129-Glatm1Kul/J**	NS	NA	NA	NA	<0.0001	<0.0001	<0.0001	<0.0001

Table S3. Genome targeting rate of the animals treated in Figure 2 was analyzed by ddPCR in genomic liver DNA preparations. The data is shown in Figure S2C.

Gene targeting rate Figure S2C P30	Gene targeting rate Liver [Mean (SD)]
**Donor WT**	0.748 (0.402)
**Donor CO02**	0.700 (0.415)
**Donor WT+Cas9**	0.897 (0.241)
**Donor CO02+Cas9**	0.220 (0.140)
**Donor WT+fludarabine**	0.772 (0.228)
**Donor CO02+fludarabine**	0.574 (0.311)

Table S4. α-Gal A enzyme activity and Lyso Gb3 (relative values) levels at P150 of the animals treated in Figure 3.

**P150 Figure 3 (P5)**	**α-Gal A enzyme activity [Mean (SD)]**				**Lyso Gb3 [relative values; Mean (SD)]**			
**Descriptive statistics**	**Plasma**	**Liver**	**Kidney**	**Heart**	**Plasma**	**Liver**	**Kidney**	**Heart**
**Donor WT+Cas9 (3.0E13 vg/kg)**	19.73 (12.68)	147.4 (135.9)	13.32 (4.69)	5.41 (1.78)	28.64 (13.15)	11.11 (8.29)	14.28 (5.36)	10.91 (3.41)
**Donor CO02+Cas9 (3.0E13 vg/kg)**	267.8 (117.5)	619.5 (194.7)	19.01 (2.37)	32.25 (14.22)	6.26 (2.39)	1.51 (0.32)	4.55 (1.50)	6.23 (2.03)
**Donor WT+Cas9 (3.0E14 vg/kg)**	2079 (1723)	2826 (3060)	158.0 (138.3)	150.5 (80.48)	3.38 (3.22)	1.19 (1.35)	1.68 (1.85)	3.02 (4.12)
**Donor CO02+Cas9 (3.0E14 vg/kg)**	4010 (1978)	9131 (7854)	8205 (5335)	541.1 (199.0)	1.33 (0.43)	0.32 (0.13)	0.51 (0.20)	0.88 (0.52)
**B6;129sf2/J**	2.42 (01.00)	8.40 (1.30)	10.00 (2.91)	4.42 (1.75)	0.29 (0.03)	0.23 (0.05)	0.40 (0.05)	0.80 (0.35)
**B6;129-Glatm1Kul/J**	0.32 (0.44)				100 (1.59)	100.0 (10.98)	100.0 (16.05)	100.0 (17.61)

Table S5. Statistical analysis (2-Way ANOVA; Tukey's multiple comparisons test) relative to α-Gal A enzyme activity and Lyso Gb3 (relative values) levels at P150 of the animals treated in Figure 3 and the data shown in Table S4.

**P150 Figure 3 (P5)**	**α-Gal A enzyme activity**				**Lyso Gb3**			
**Tukey's multiple comparisons test**	**Plasma**	**Liver**	**Kidney**	**Heart**	**Plasma**	**Liver**	**Kidney**	**Heart**
**Donor WT+Cas9 (3.0E13 vg/kg) vs. Donor CO02+Cas9 (3.0E13 vg/kg)**	NS	NS	NS	NS	<0.0001	NS	NS	NS
**Donor WT+Cas9 (3.0E13 vg/kg) vs. Donor WT+Cas9 (3.0E14 vg/kg)**	0.0398	NS	NS	NS	<0.0001	NS	NS	NS
**Donor WT+Cas9 (3.0E13 vg/kg) vs. Donor CO02+Cas9 (3.0E14 vg/kg)**	<0.0001	0.0074	0.0001	<0.0001	<0.0001	NS	NS	NS
**Donor WT+Cas9 (3.0E13 vg/kg) vs. B6;129sf2/J**	NS	NS	NS	NS	<0.0001	NS	NS	NS
**Donor WT+Cas9 (3.0E13 vg/kg) vs. B6;129-Gla$_{tm1Kul}$/J**	NS	NA	NA	NA	<0.0001	<0.0001	<0.0001	<0.0001
**Donor CO02+Cas9 (3.0E13 vg/kg) vs. Donor WT+Cas9 (3.0E14 vg/kg)**	NS	NS	NS	NS	NS	NS	NS	NS
**Donor CO02+Cas9 (3.0E13 vg/kg) vs. Donor CO02+Cas9 (3.0E14 vg/kg)**	0.0002	0.0115	0.0001	<0.0001	NS	NS	NS	NS
**Donor CO02+Cas9 (3.0E13 vg/kg) vs. B6;129sf2/J**	NS	NS	NS	NS	NS	NS	NS	NS
**Donor CO02+Cas9 (3.0E13 vg/kg) vs. B6;129-Gla$_{tm1Kul}$/J**	NS	NA	NA	NA	<0.0001	<0.0001	<0.0001	<0.0001
**Donor WT+Cas9 (3.0E14 vg/kg) vs. Donor CO02+Cas9 (3.0E14 vg/kg)**	NS	NS	0.0002	<0.0001	NS	NS	NS	NS
**Donor WT+Cas9 (3.0E14 vg/kg) vs. B6;129sf2/J**	0.0376	NS	NS	NS	NS	NS	NS	NS
**Donor WT+Cas9 (3.0E14 vg/kg) vs. B6;129-Gla$_{tm1Kul}$/J**	0.0373	NA	NA	NA	<0.0001	<0.0001	<0.0001	<0.0001
**Donor CO02+Cas9 (3.0E14 vg/kg) vs. B6;129sf2/J**	<0.0001	0.0065	0.0001	<0.0001	NS	NS	NS	NS
**Donor CO02+Cas9 (3.0E14 vg/kg) vs. B6;129-Gla$_{tm1Kul}$/J**	<0.0001	NA	NA	NA	<0.0001	<0.0001	<0.0001	<0.0001
**B6;129sf2/J vs. B6;129-Gla$_{tm1Kul}$/J**	NS	NA	NA	NA	<0.0001	<0.0001	<0.0001	<0.0001

Table S6. Genome targeting rate of the animals treated in Figure 3 was analyzed by ddPCR in genomic liver DNA preparations. The data is shown in Figure S3D.

**Vector and dose**	**Integration rate (Mean (SD)]**
Donor WT+Cas9 (Low)	0.2120 (0.1154)
Donor CO02+Cas9 (Low)	0.8500 (0.3429)
Donor WT+Cas9 (High)	8.528 (4.803)
Donor CO02+Cas9 (High)	8.098 (2.233)

Table S7. α-Gal A enzyme activity and Lyso Gb3 levels at P150 of the animals treated in Figure 4.

P150 Figure 4 (P5)	α-Gal A enzyme activity [Mean (SD)]				Lyso Gb3 [ng of lysoGb3/ml of plasma or mg of tissue; Mean (SD)]			
**Descriptive statistics**	**Plasma**	**Liver**	**Kidney**	**Heart**	**Plasma (ng/ml)**	**Liver (ng/mg)**	**Kidney (ng/mg)**	**Heart (ng/mg)**
**Donor CO02**	16.59 (5.21)	33.90 (7.47)	12.50 (2.85)	9.38 (1.77)	65.11 (23.18)	0.60 (0.22)	0.32 (0.10)	0.093 (0.038)
**Donor CO02+Cas9**	2575 (1500)	68.93 (16.20)	29.38 (21.35)	34.83 (11.19)	16.78 (4.00)	0.14 (0.10)	0.07 (0.03)	0.060 (0.049)
**Donor CO02+Drug**	29.63 (8.38)	32.11 (8.34)	11.00 (1.38)	9.99 (2.99)	53.46 (19.22)	0.62 (0.41)	0.23 (0.12)	0.116 (0.026)
**B6;129sf2/J**					0.28 (0.03)			
**B6;129-Glatm1Kul/J**	0.90 (0.06)	11.36 (0.73)	11.60 (0.69)	4.51 (0.25)	354.4 (43.09)	9.44 (2.82)	2.02 (0.33)	0.748 (0.131)

Table S8. Statistical analysis (2-Way ANOVA; Tukey's multiple comparisons test) relative to α-Gal A enzyme activity and Lyso Gb3 (relative values) levels at P150 of the animals treated in Figure 4 and the data shown in Table S7.

P150 Figure 4 (P5)	α-Gal A enzyme activity				Lyso Gb3			
**Tukey's multiple comparisons test**	**Plasma**	**Liver**	**Kidney**	**Heart**	**Plasma**	**Liver**	**Kidney**	**Heart**
B6;129-Gla$_{tm1Kul}$/J vs. Donor CO02	NS	0.048	NS	NS	0.0429	0.0003	<0.0001	<0.0001
B6;129-Gla$_{tm1Kul}$/J vs. DonorCO02+Drug	NS	0.0516	NS	NS	0.0064	0.0002	<0.0001	<0.0001
B6;129-Gla$_{tm1Kul}$/J vs. DonorCO02+Cas9	0.0068	0.0004	NS	0.0003	NS	0.0005	<0.0001	<0.0001
B6;129-Glatm1Kul/J vs. B6;129sf2/J					0.0017			
Donor CO02 vs. DonorCO02+Drug	NS	NS	NS	NS	NS	NS	NS	NS
Donor CO02 vs. DonorCO02+Cas9	0.0070	0.0087	NS	0.0011	NS	NS	NS	NS
AAV8 Donor CO02 vs. B6;129sf2/J					NS			
DonorCO02+Drug vs. DonorCO02+Cas9	0.0072	0.0047	NS	0.0009	NS	NS	NS	NS
AAV8 Donor CO02+DRUG vs. B6;129sf2/J					0.0388			
AAV8 Donor CO02+Cas9 vs. B6;129sf2/J					NS			

Table S9 Statistical analysis (1-Way ANOVA; Tukey's multiple comparisons test) relative to latency to heat (hot plate test) of the animals treated in Figure 4 and the data shown in Figure 5. One Way ANOVA P=0.0007

**Hot plate Figure 5 (P5)**	
**Tukey's multiple comparisons test**	**P value**
B6;129-Glatm1Kul/J vs. Donor CO02+Cas9	<0.0001
B6;129-Glatm1Kul/J vs. Donor CO02+Drug	0.0006
B6;129-Glatm1Kul/J vs. Donor CO02	0.0001
B6;129-Glatm1Kul/J vs. B6;129sf2/J	<0.0001
Donor CO02+Cas9 vs. Donor CO02+Drug	NS
Donor CO02+Cas9 vs. Donor CO02	NS
Donor CO02+Cas9 vs. B6;129sf2/J	NS
Donor CO02+Drug vs. Donor CO02	NS
Donor CO02+Drug vs. B6;129sf2/J	0.0210
Donor CO02 vs. B6;129sf2/J	NS

Supplemental figures

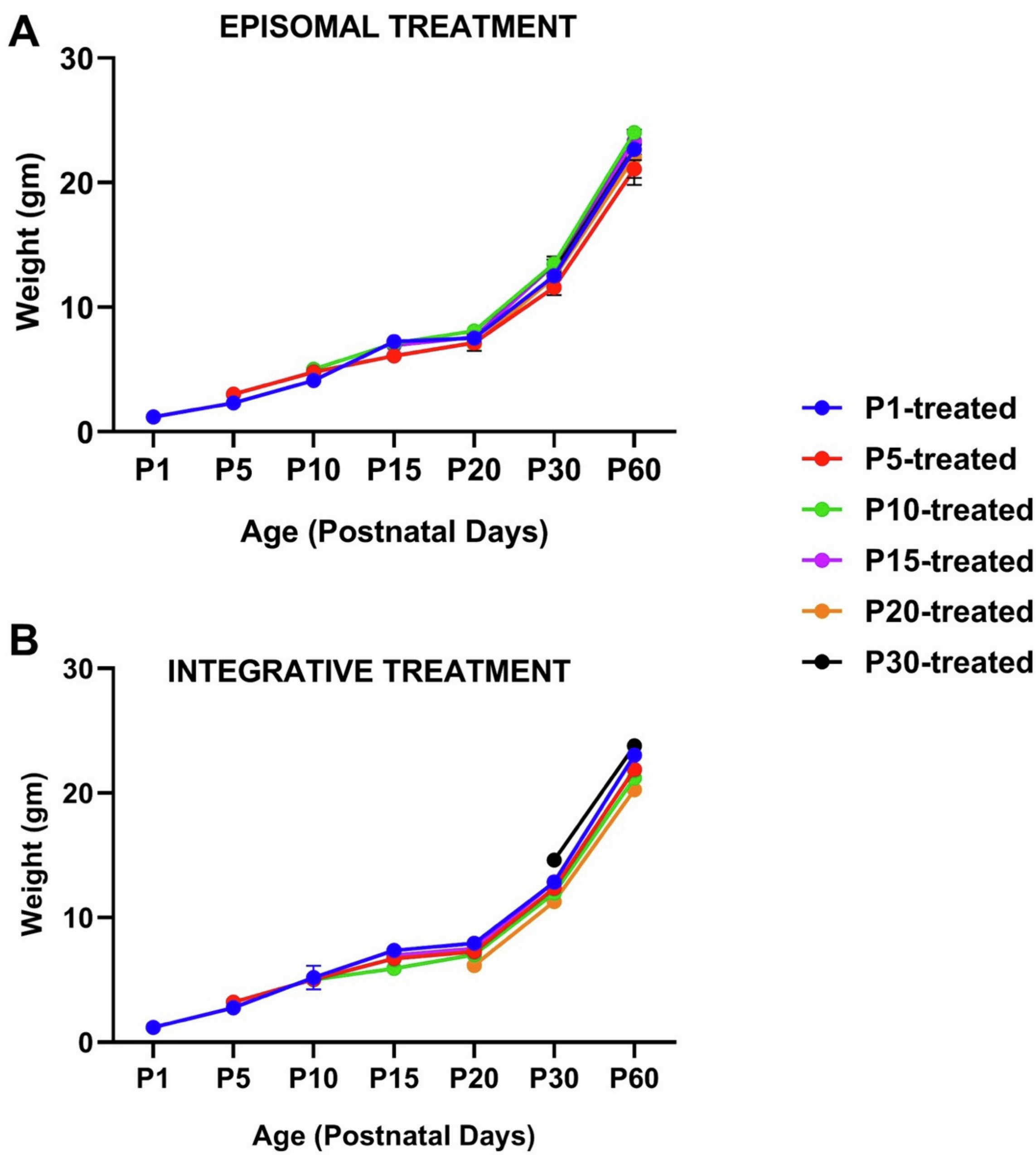

Figure S1. Growth curve. The animals of the episomal (A) and integrative (B) treatments groups shown in Figure 1 were weighted over the course of the experiment. Mixed-effects model (REML), NS.

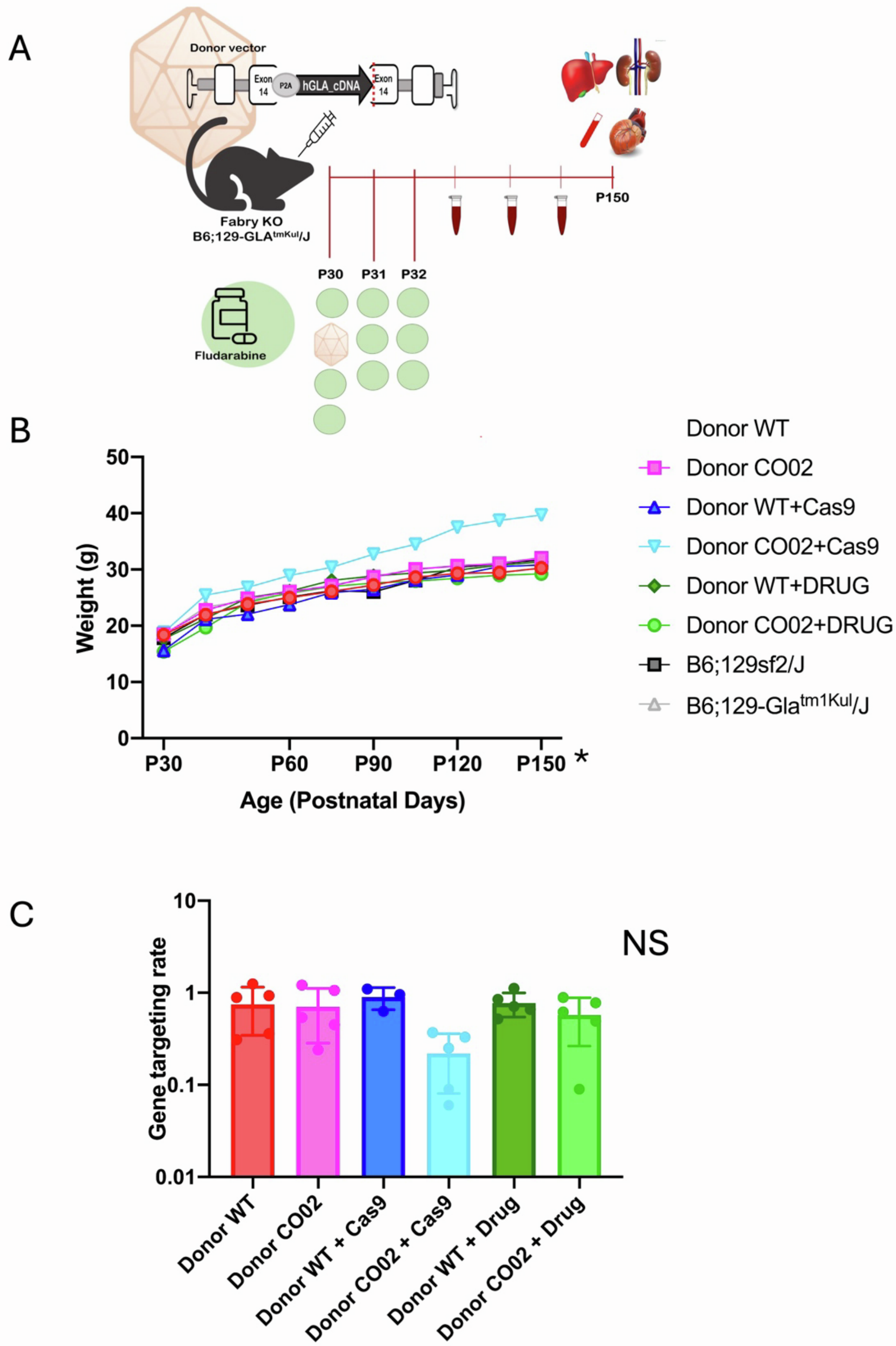

Figure S2. Genome targeting treatment of juvenile P30 Fabry mice. A) Fludarabine administration. Experimental scheme. Fludarabine was administered for three consecutive days (P30, P31, and P32; three doses per day). The AAV donor vector was administered on P30. Blood was collected at different timepoints, and liver, heart, and kidney were collected at sacrifice (P150); **B)** Growth curve. Mixed-effects model (REML), time: P<0.0001; Treatment, P<0.0001, time x treatment, P,0.0001; *, Donor CO02+Cas9 different to the other groups; **C)** Quantitative ddPCR was done with genomic DNA extracted from liver homogenates. Each dot represents a single animal. One way ANOVA, P=0.0529, NS.

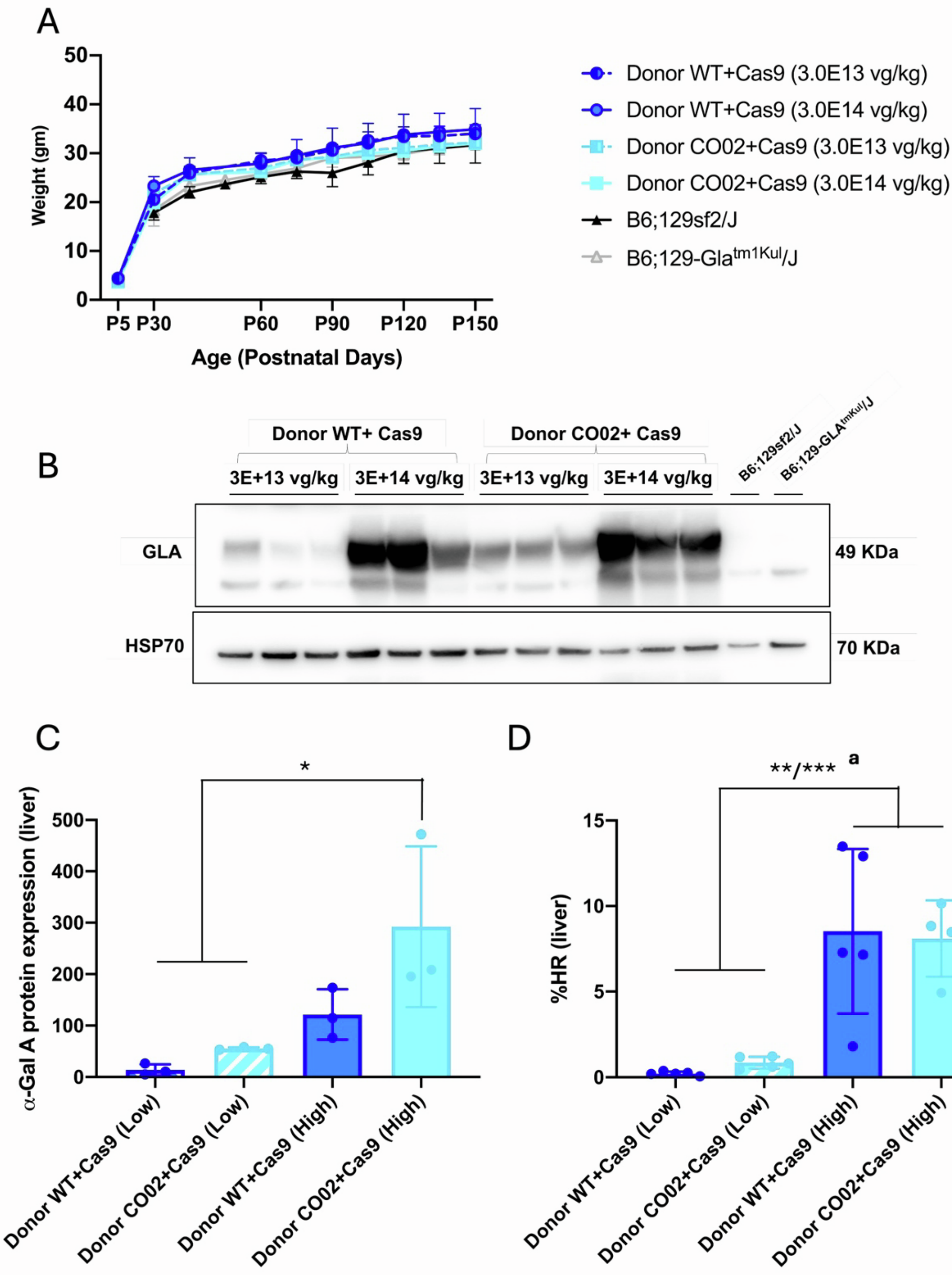

Figure S3. Genome targeting treatment of newborn P5 Fabry mice. A) Growth curve. Two-Way RM Anova: time x treatment P<0.0001; time P<0.0001; Treatment P<0.0001; **B)** Western blot assay of protein extracts from liver homogenates, using anti-a-Gal A specific antibody. HSP70 was used as control. **C)** The bar graph shows the quantification of the blot of Panel B. Each dot corresponds to a single animal. Two-way ANOVA (P<0.05) with multiple comparisons, *, P<0.05; **D)** Quantitative ddPCR was done with genomic DNA extracted from liver homogenates. Each dot represents a single animal. Two-way ANOVA, P<0.05; multiple comparisons, **, P<0.01, ***, P<0.001. [a], Donor WT+Cas9 (low) vs. Donor CO02+Cas9 (high), P<0.001.

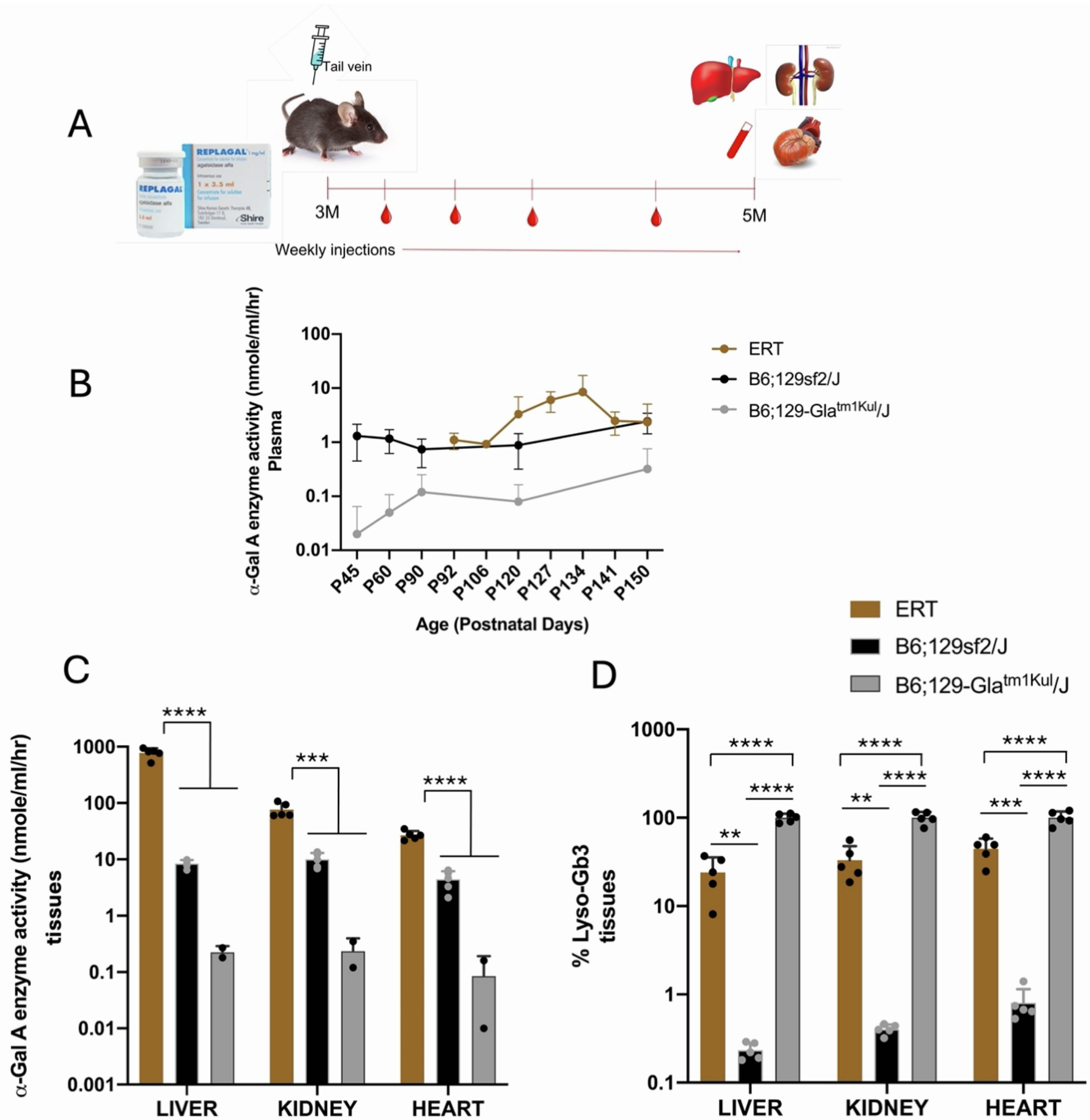

Figure S4. Treatment of Fabry mice with ERT. A) Experimental scheme. Male B6;129-*Gla*$^{tm1Kul/J}$ mice were dosed weekly with ERT (Replagal). Blood was collected at different timepoints, and liver, heart, and kidney were collected at sacrifice (P150). **B)** α-Gal A enzyme activity was measured in blood plasma collected at different time points. The graph shows the values of untreated B6;129-*Gla*$^{tm1Kul/J}$ mice and WT controls. The graph shows the mean of the activity (nmoles/ml/hr) at each time point. **C, D)** Bar graph representation of α-Gal A enzyme activity (C) and lyso-Gb3 accumulation (D) at P150 in liver, kidney, and heart. Each dot represents a single animal. One-Way ANOVA, P<0.0001; Tukey's multiple comparisons, **, P<0.01; ***, P<0.001; ****, P<0.0001.

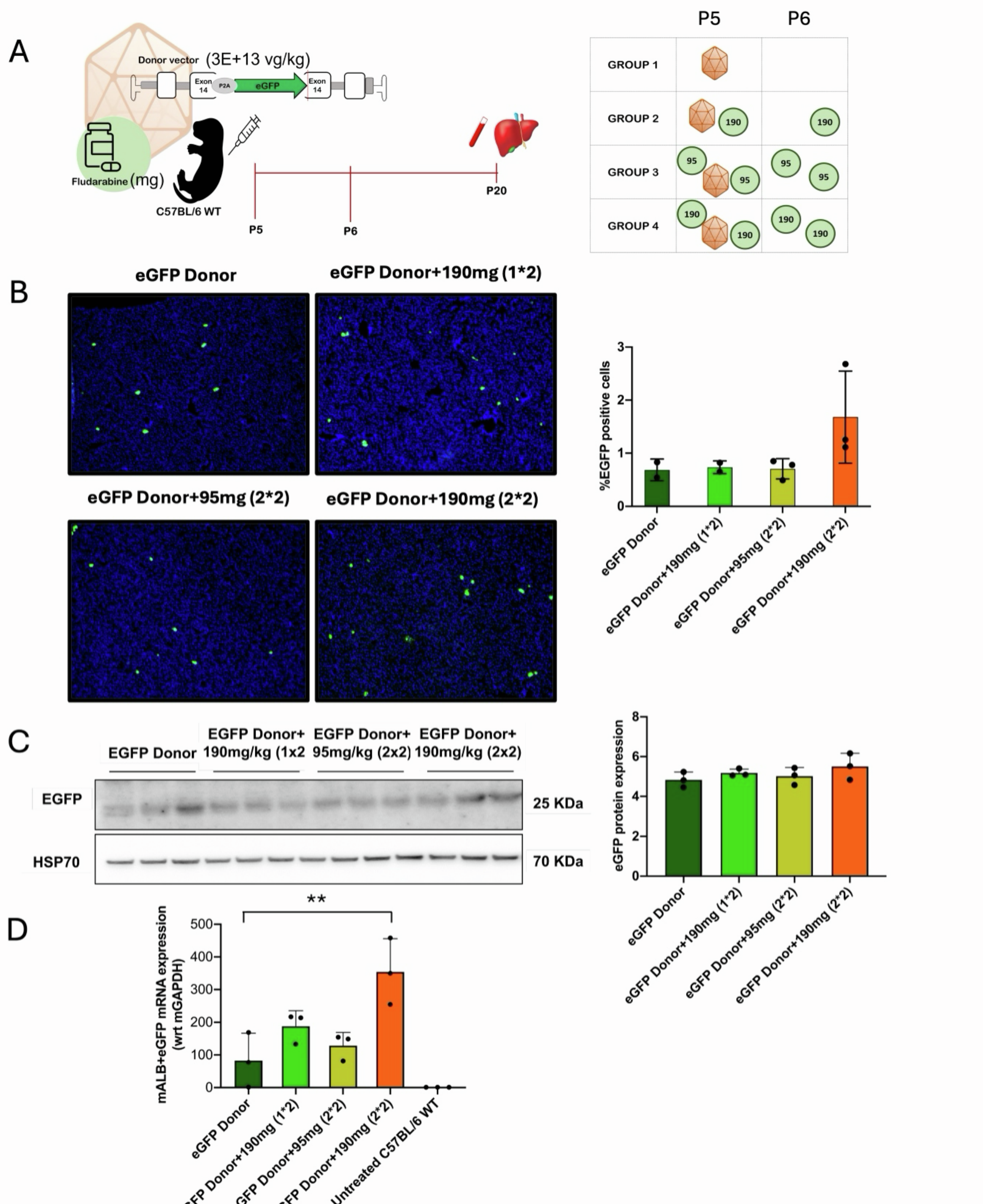

Figure S5. Genome targeting treatment and fludarabine administration to newborn P5 WT mice A) Experimental scheme. Newborn P5 WT male mice were administered with fludarabine and eGFP - donor vector as indicated in the table (right). The numbers inside the green circles indicate the dose of fludarabine (mg/kg); **B)** Immunofluorescence of representative liver sections of the different treated animals (left). (left). The housekeeping gene HSP70 was used as internal control. Quantification of the eGFP WB (right). Each dot corresponds to a single animal. One-Way ANOVA, NS; **D)** qRT-PCR analysis of the chimeric albumin-eGFP mRNA from the liver of treated mice (normalized to mGAPDH). One-Way ANOVA, P<0.001; Multiple comparisons, **, P<0.01.

A

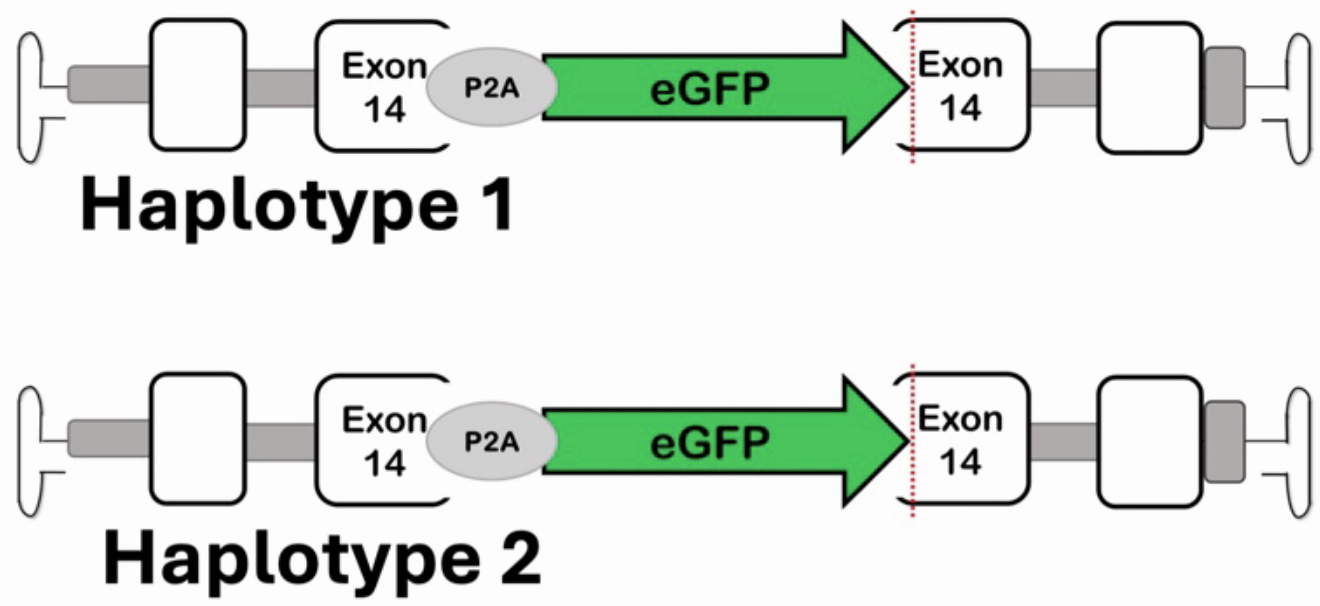

Position	ID	REF	ALT	HAP1	HAP2	HAP3	HAP4	HAP5	HAP6	HAP7
74285239	rs962004	C	T	T	C	C	C	C	T	C
74285552	rs4076	A	G	A	G	A	A	A	G	G
74285567	rs962005	C	A	C	A	C	C	C	A	A
74285758	rs2236766	G	T	G	T	T	G	G	T	T
74285823	rs2236767	G	A	G	A	G	G	G	A	A
74287403	rs4429703	T	C	C	T	T	C	T	T	C
Frequency				50.14%	44.69%	2.15%	1.51%	1.37%	0.09%	0.05%

B

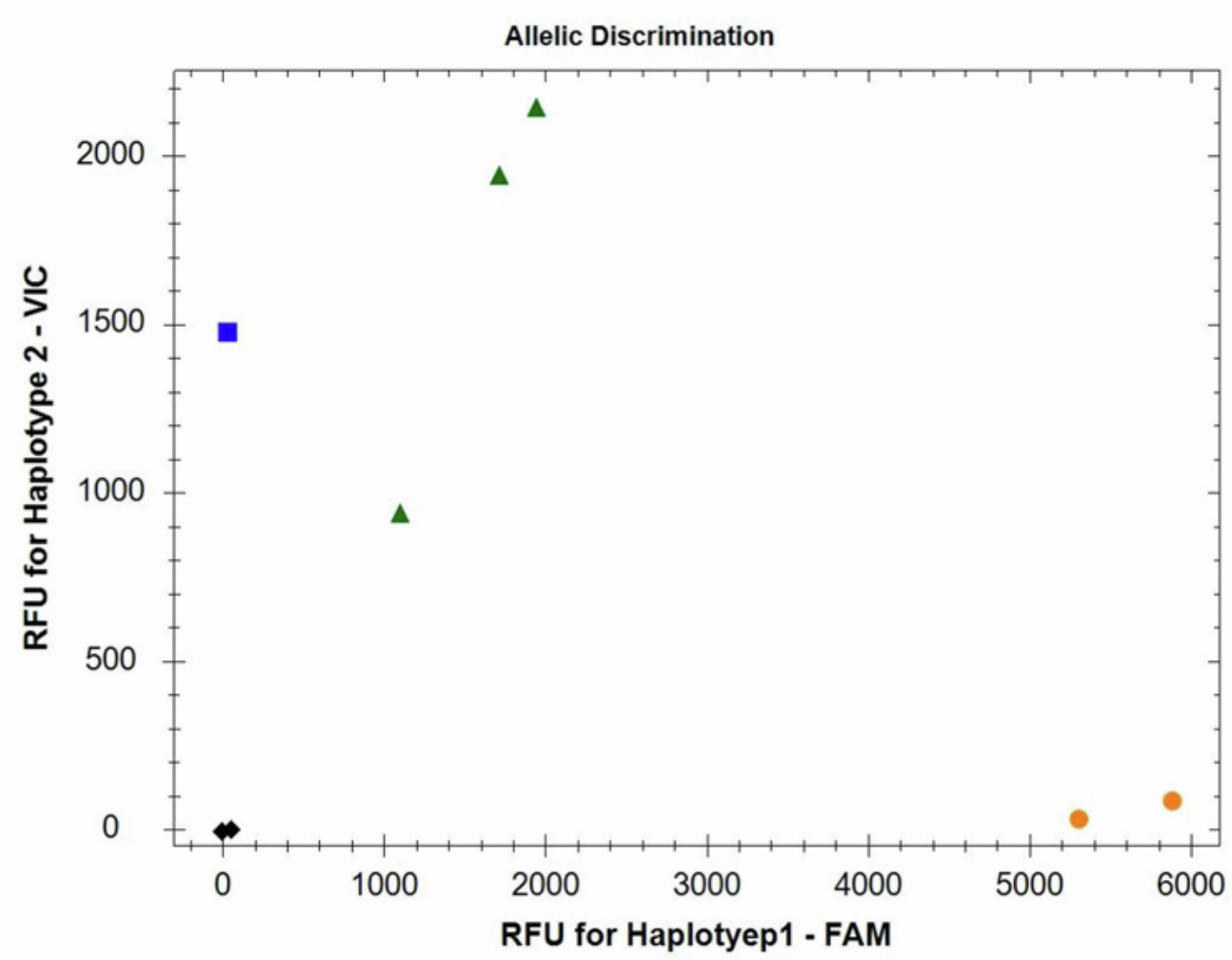

Haplotype	Fluorophore	Allele	Sequence SNP
Haplotype 1	FAM	Allele 1	TTGTTGAGCTT
Haplotype 2	VIC	Allele 2	TTGTTGAGCTC

SAMPLE		SAMPLE TYPE	GENOTYPE
**HuH7**	■	Human hepatocyte cell line	Haplotype 2
**Sample A**	●	Human blood	Haplotype 1
**Sample B**	▲	Human blood	Heterozygote
**Sample C**	▲	Human blood	Heterozygote
**Sample D**	▲	Human blood	Heterozygote
**Water**	◆	Negative control	NA

Figure S6. Genome targeting in human cells. Taq-Man PCR detection method of albumin haplotypes. A) Scheme of the donor vectors (top) containing the two most frequent albumin haplotypes present in the human population (bottom, Ref. 47). **B)** Taq-Man PCR of genomic DNA from a hepatoma cell line and human blood samples.
